# SPECHT: Self-tuning Plausibility based object detection Enables quantification of Conflict in Heterogeneous multi-scale microscopy

**DOI:** 10.1371/journal.pone.0276726

**Published:** 2022-12-29

**Authors:** Ben Cardoen, Timothy Wong, Parsa Alan, Sieun Lee, Joanne Aiko Matsubara, Ivan Robert Nabi, Ghassan Hamarneh

**Affiliations:** 1 Medical Image Analysis Laboratory, School of Computing Science, Simon Fraser University, Burnaby, British Columbia, Canada; 2 Department of Cellular and Physiological Sciences, Life Sciences Institute, University of British Columbia, Vancouver, British Columbia, Canada; 3 Department of Ophthalmology and Visual Sciences, Eye Care Centre, University of British Columbia, Vancouver, British Columbia, Canada; 4 Mental Health & Clinical Neurosciences, School of Medicine, University of Nottingham, Nottingham, United Kingdom; 5 School of Biomedical Engineering, University of British Columbia, Vancouver, British Columbia, Canada; University of Houston, UNITED STATES

## Abstract

Identification of small objects in fluorescence microscopy is a non-trivial task burdened by parameter-sensitive algorithms, for which there is a clear need for an approach that adapts dynamically to changing imaging conditions. Here, we introduce an adaptive object detection method that, given a microscopy image and an image level label, uses kurtosis-based matching of the distribution of the image differential to express operator intent in terms of recall or precision. We show how a theoretical upper bound of the statistical distance in feature space enables application of belief theory to obtain statistical support for each detected object, capturing those aspects of the image that support the label, and to what extent. We validate our method on 2 datasets: distinguishing sub-diffraction limit caveolae and scaffold by stimulated emission depletion (STED) super-resolution microscopy; and detecting amyloid-*β* deposits in confocal microscopy retinal cross-sections of neuropathologically confirmed Alzheimer’s disease donor tissue. Our results are consistent with biological ground truth and with previous subcellular object classification results, and add insight into more nuanced class transition dynamics. We illustrate the novel application of belief theory to object detection in heterogeneous microscopy datasets and the quantification of conflict of evidence in a joint belief function. By applying our method successfully to diffraction-limited confocal imaging of tissue sections and super-resolution microscopy of subcellular structures, we demonstrate multi-scale applicability.

## Introduction

Fluorescence microscopy is a robust experimental tool for the study of biological samples. Applications range from micrometer-scale labelling of tissues to super-resolution nanometer-scale analysis of molecular components of cells [1]. In such experiments, a common goal is to detect and describe differences between the experimental groups evidenced by the differences in their images. This problem can be framed as weakly supervised object detection (WSOD), a challenging task of locating objects in images given only image-level labels. More formally, given a set of microscopy images {I} from different groups (label *L*: e.g. normal, control, treated, infected, mutation, wild type), we want to identify the parts of the image that show evidence for its label *L*, and quantify the confidence in the identification. In fluorescence microscopy studies the challenge is magnified by the heterogeneity in the images from using multiple samples, diverse fluorescent markers often imaged in parallel in different fluorescent channels, and various microscopes and operators. There is a clear need for fluorescence image analysis methods that adapt dynamically to changing imaging conditions.

Unlike the classical WSOD problem statement where an object in nature is either, say, a cat or horse, objects identified using SPECHT (Self-tuning Plausibility Based Object Detection Enables Quantification of Conflict in Heterogeneous Multi-scale Microscopy) can assign support for multiple labels to a single object. To overcome per image variation in acquisition, we introduce an adaptive approach that detects objects by thresholding the Laplacian of each image, using its kurtosis to ensure the threshold scales with the distribution of each specific image. Using belief theory we then assign to each identified object the support or numerical evidence it has for a set of labels, and introduce how belief calculus can offer the user interpretable information on the conflict and agreement of composite models learned on heterogeneous datasets. Here, we apply SPECHT to two use cases of distinct scale: identification of intermediate stages in the construction of more complex subcellular structures using super-resolution microscopy and detection of a gradual pathology from fluorescent confocal microscopy images of tissue sections.

First, caveolae, subcellular structures composed of CAV1 protein complexes, are ∼100 nm invaginations in the cell membrane with a varied spectrum of functions [2]. CAV1 forms non-caveolar scaffolds (SC), including 8S oligomers that combine to form larger non-caveolar hemispherical scaffolds as well as caveolae [1, 3–6]. In the absence of CAVIN1, CAV1 forms non-caveolar scaffold structures that contain few CAV1 molecules and those can only be distinguished from caveolae by super-resolution microscopy [1]. Caveolae flattening functions as a membrane buffer, protecting cells from membrane breakage in response to mechanical stress, and scaffolds have been shown to be pro-metastatic in prostate cancer [7, 8]. In fixed cells, superresolution network analysis identifies individual scaffolds into separate sub-types whose modular similarity suggests that smaller scaffolds combine to form larger scaffolds and caveolae [1, 5]. This represents an example of hierarchical modelling in cell biology in which a larger modular protein structure is composed of smaller sub-units, with both having distinct functions and properties, e.g. the smaller structure(s) can exist as independent, functional units [5]. We show that application of SPECHT to an alternate super-resolution microscopy approach, STED [9], amenable to high speed live cell imaging [10], is capable of distinguishing these sub-diffraction limited sub-cellular structures.

Second, identification and quantification of amyloid-*β* (A*β*) deposits in the retina in relation to Alzheimer disease (AD) is an open research problem [11]. In previous studies using confocal microscopy on post-mortem donor tissues, retinal A*β* quantification was performed manually by blinded raters [12] or semi-automatically with manual segmentation [13]. The resulting measurement of retinal A*β* would be tested for its relationship with age, AD neuropathology, retinal regions, and other measures of interest. As the scarcity of postmortem retinal tissues from neuropathologically confirmed AD donors often limits the size of such data, variability from acquisition and manual raters can affect the quantification of retinal A*β* and poses a challenge to achieving statistical significance.

Finally, when we present the detected and identified objects to the domain expert, be they cell biologist or medical practitioner, we want to be able to report how ‘sure’ our method is in its computation. With biomedical data typically scarce and acquired with differently configured devices, we would like to have a method that can work across these datasets, to maximize reuse and reproducibility. If models computed on different dataset disagree in the identification of objects, we want to report a mathematically grounded quantification of this disagreement or conflict.

### Problem statement

We aim to model a function D that identifies subsets of pixels (objects *o*) of the image and a function S_*L*_ that assigns to each object the statistical support, or evidence *e*, for the image label *L*. Statistical support is the numerically quantified evidence distilled by a statistical (learning) method from feature descriptors and their distribution.

$$\begin{array}{c} \text{D}:\text{I}\mapsto o\;\;|\;o\subset \text{I}. \end{array}$$ (1)

$$\begin{array}{c} {\text{S}}_{L}:\left(o\to L\right)\mapsto e\;\;|e\in \left[0,1\right],\;o\subset \text{I},L\in L. \end{array}$$ (2)

The notation f: *A* ↦ *B* indicates that the function f has domain A and range B. In the remainder of this work, we use the notation *o* → *L* for the proposition that the object o supports the label *L*, and S_*L*_ = S(*o* → *L*) is the function S assigning a continuous (*e* ∈ [0, 1]) support value to the proposition in the context of belief theory [14], a generalization of probability functions. In other words, if an object o has high support for image label L, it can be considered likely or typical to appear in images with label L. A ‘frame of discernment’ Θ={o→L|o⊂I,L∈L} is the set of all sources of evidence for the image *I* and label-space L. Labels can be hierarchically organized; for example the label ‘disease’ can be further specialized into specific sub-conditions, or sub-labels. An advantage of using belief theory to model this problem is that it does not require each proposition to be a singleton. Belief theory allows us to specify only the support we can compute. If we do not have measurable evidence to compute the support an object has for a specific sub-label, then we do not need to assign it an arbitrary evidence score, as long as we can assign some evidence to its superset. This enables us to extend our model as more information becomes available, and lends itself to hierarchical or nested, and often modular label spaces. A hierarchical or nested label space L is where subsets of labels have a label. For example the label ‘Disease’ (D) can encompass both ‘Alzheimer’ (A) and ‘Parkinson’ (P): A,D,P∈L,D⊂L,D={A,P}. A modular label space is a specific type of hierarchical label space that models a structure (S), comprised of parts with specific labels (*P*_1_, *P*_2_, …, *P*_*n*_), as a subset: $S,P_{i}\in L,S\subset L,S=\left\{P_{i}|\;\forall i=1,..,n\right\}$. In our data, caveolae (C), are constructed by aggregating different types of scaffolds (S): C,S∈L,C={S}. With our use cases featuring nested label spaces, our choice of selecting belief theory as a framework is justified.

### Related work

State-of-the-art methods tackling problems closest to our problem statement are broadly divided into (i) joint segmentation and classification, (ii) interpretable deep learning, (iii) multiple instance learning (MIL), and (iv) weakly supervised object detection and localization (WSOD/L). However, each presents deficiencies with respect to application of the method across data sets as well as to object classification.

### Joint or hybrid segmentation and classification

In joint or hybrid segmentation and classification learning, higher accuracy can be obtained in the classification task, while class specific priors can then be leveraged to improve segmentation [15]. Similar approaches have been applied to chromosome microscopy [16], breast biopsy [17], fundus images[18], and histopathology [19] to name a few. However, typically they require object level annotations, which we do not have. In contrast with their object level classification, we aim to capture a gradual transition between classes, e.g. discrete versus continuous object level annotations. In addition, they do not always provide a theoretical upper bound to the support assigned to each segment, meaning that in principle the label assigned to the object can take on extreme values not warranted by the data. It is unclear how to apply the same method across heterogeneous datasets, or to quantify conflict between models learned from such datasets.

### Explainable AI

In explainable AI neural networks can, for example, produce the regions of the image that provide the most decisive information supporting the predicted image level label, are covered in more detail in recent reviews [20, 21]. Recently, these approaches are accompanied by domain fusion, for example augmenting MR images of Alzheimer’s patients with their meta-data to learn the MRI signatures of Alzheimer’s disease [22], or fusing diagnostic reports with image data [23] to offer interpretable improved diagnosis. While in such approaches the support each region has for a single label is found, it is not optimized to split those regions into smaller distinct objects. In addition, there are no non-trivial (0,1) bounds on the support that each object is assigned, potentially inducing high uncertainty. Filtering the attention maps [24] to obtain a more precise delineation of which regions of an image support a label is one direction that aims to close the gap towards granular object detection folded into interpretable AI. Recent work adds the computation of uncertainty to the ‘importance’ of features in interpretable AI [25], however, the output for image features is still region based, rather than object based.

### Multiple instance learning

In MIL terminology, a label exists for a ‘bag’ of instances. The ‘bag’ can refer to the image, where instances would be objects in the image. The standard MIL model has it that all bags with label *L*^−^ only contain instances with label *L*^−^. Bags with label *L*^+^ contain instances with at least one instance (‘witness’) with label *L*^+^. Alternatively, the MIL formulation can be adapted to learn the distribution of labels over a bag [26]. MIL has been adopted successfully for microscopy-specific tasks such as classifying and segmenting cells [27, 28] with recent reviews [29, 30] detailing the different approaches. We are not aware of MIL methods that incorporate the explicit encoding of (conflicting) evidence and uncertainty in the context of evidence theory, nor do MIL approaches feature a theoretical bound on the support for each observed instance.

### Weakly supervised object detection and localization

A complete review of WSOD/L methods has been presented recently [31], and is indicative by the sharp rise in deep learning based WSOD/L approaches. However, subtle yet critical differences are present between our problem statement and the problem statement addressed in WSOD/L methods. Uncertainty, in our context, is interpreted as the margin of error in assigning a certain support to an object. In WSOD/L the uncertainty refers to the noise or variability induced by the human made image level label annotation [31], domain shift, image noise, variability in object appearance, or imprecise localization annotations (e.g. size of enclosing bounding box). In our use case we have no ground truth localizations or bounding boxes to compare against. Deep learning based WSOD/L methods can be sensitive to small dataset sizes with long tail distributions, which, unfortunately, is the case for both our datasets and is common in biomedical data for scientific discovery. We do have the advantage of leveraging acquisition specific information to inform the object detection stage. WSOD/L methods do, to the best of our knowledge, not quantify or report the evidence based on object features in the end results, nor do they feature joint models across heterogeneous datasets, which we require. Interestingly, it has been suggested that such refining of evidence is a key attribute of biological vision systems [32], and likely to drive adoption towards reinforcement learning based methods for WSOD/L. Finally, the detection of ‘small’ objects, typically referenced as <16×16 pixels [33], is still an open problem in deep learning based approaches for WSOD/L [33, 34]. Part of this difficulty stems from the limited potential for discriminative deep features such small objects offer. Worse, this problem becomes exponentially harder as object size decreases, in other words there is an explicit bias towards object size in function of recall, which can compromise biological discovery. In natural images, small object detection by remote sensing, for example by unmanned arial vehicles (UAV), was recently mitigated by exploiting tracking of registered objects over time [33, 34] in a supervised learning setting. Similarly, exploiting global information across the image [35] was shown to improve results. However, feature extraction becomes even more challenging when the source images are no longer natural images. For instance, single-channel superresolution microscopy, at the limit of physical observation, often heavily perturbed by both acquisition and semantic noise. In addition, here WSOD/L is leveraged with the end goal of discovery, without object level ground truth, requiring an unbiased result. In superresolution microscopy adding a temporal dimension invariably leads to compromising spatial accuracy, making the temporal tracking mitigation not a viable approach. The problematic ‘small’ category object designation in natural RGB images of 16×16 pixels in our application results in structures of a diameter of 452Â nm^2^ being designated as small enough to be problematic in classification, yet the largest structure of interest, caveolae, have a diameter of 100nm at most [36]. Consequently, this necessitates an approach that does not rely on deep learning features to work reliably.

The closest related work to ours for the detection of caveolae is Label2Label [37], where a network is trained in a supervised setting to reconstruct caveolae, however, this approach learned to ignore, as part of cytosolic background [38], the key components of caveolae: scaffolds. Previous work [4] shows that the CAV1 structures in the membrane are compromised of no less than 4 non-crisp classes of CAV1 structures, with caveolae only accounting for 20% [1, 5]. Indeed, the difficulty of recovering caveolae and smaller composite structures in STED was the motivation for the introduction of ‘line-switching 2-color’ for triple-relaxation (T-Rex) STED [39], which jointly optimizes 2-color acquisition as a remediating step. However, this still requires adaptive unsupervised object detection to accurately identify the composite nature of the recovered structures. In contrast to the Label2Label approach, we show that it is possible to identify and differentiate not just caveolae, but also scaffolds in 2D STED, without requiring improved acquisition. In addition, we show that our usage of belief theory allows the combination of models constructed on datasets acquired by different operators in an elegant mathematical framework, including the capture of the uncertainty and conflict between the interpretable evidence each model has for a weak label.

Finally, we show that the open problem of identification of amyloid-*β* plaques in fluorescence confocal microscopy of retinal tissue conditional on Alzheimer disease features identical obstacles to CAV1 detection in STED. We show that our method can work on both, without specializing on either. In immunohistochemistry, measuring fluorescence signal in confocal microscopy images has largely relied on manual annotation or traditional image intensity thresholding techniques such as histogram adaptation or clustering, with ImageJ-based Fiji being a widely used software for this purpose [40, 41]. The small areas, granular distribution, unclear signal, noise, and background borders of the fluorescence signal, and the variability in the image quality, acquisition protocols, and staining and sample quality present inherent challenges for both approaches, with the latter often employing semi-automated techniques for annotation quality control [42]. In recent years, several studies have investigated deep-learning for fluorescence image analysis, focusing mainly on supervised image classification or segmentation of specific cellular or subcellular structures [43–46]. However, manual feature annotation in fluorescence images is well-known to be highly subjective [47, 48]. The variable quality of annotation, in part caused by low or variable visibility of the fluorescent targets, can result in the DL model failing to train properly or produce consistent annotations on new data [42, 49, 50]. In this work, we demonstrate how SPECHT identifies fluorescence signal of biological interest given only the image-level label, and, moreover, robustly performing this task given two sets of images acquired using different microscopes by different researchers.

### Proposed contribution

We introduce here SPECHT for object detection and evidence-based object labelling. SPECHT involves two stages:

- Adaptive and self-tuning object detection using the kurtosis of the Laplacian to match distributions across channels for fluorescence microscopy.
- Belief theory-based labelling to quantify the non-crisp evidence each identified object has for a set of image-level labels.

**Fig 1:**
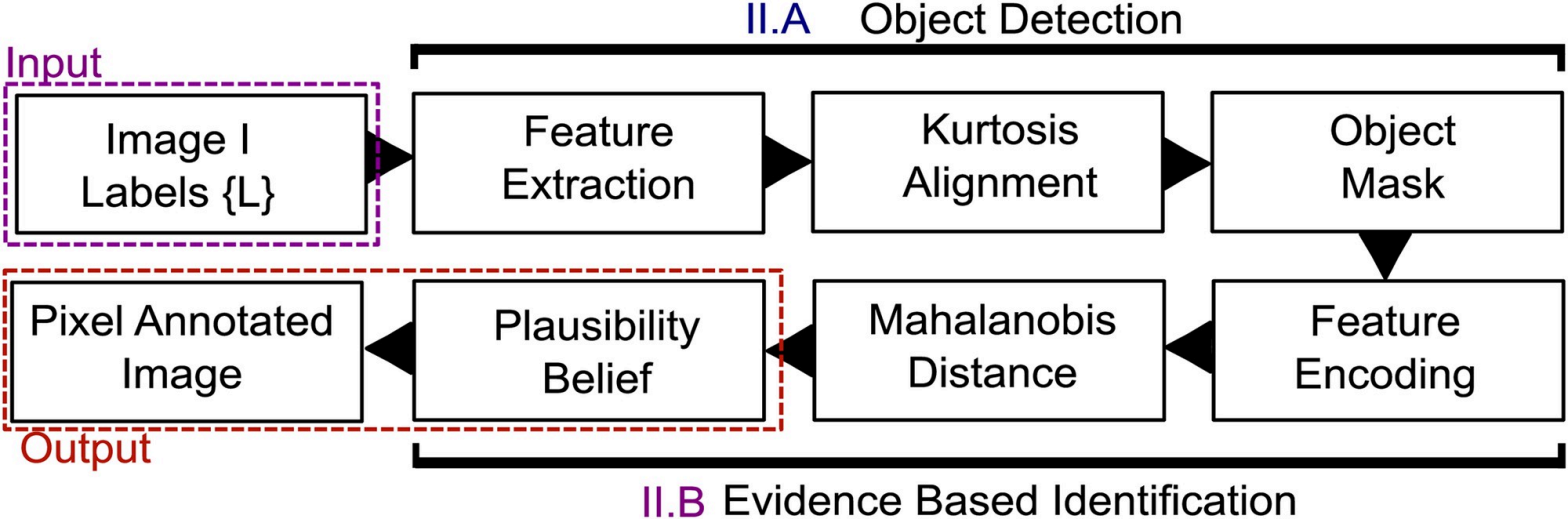
SPECHT algorithm. Adaptive identification of subcellular structures in superresolution microscopy in tandem with belief based labelling of each object’s support for the cell level genotype. Each image can have a set of labels, SPECHT then computes for each identified object o the support it has per individual label.

Use of kurtosis enables estimation of algorithm-specific parameters consistently across heterogeneous data in the absence of object-level annotation, providing a novel, self-tuning, and robust framework for analyzing images. The class of problems we address here is identification of fluorescently labelled structures from background and fuzzy classification of these structures from each other. The algorithm is illustrated in Fig 1A.

We use SPECHT to, first, identify and distinguish sub-diffraction limit (<100 nm diameter) caveolin-1 (CAV1) domains using STED [9] super-resolution microscopy. Next, we show that SPECHT can automatically distinguish amyloid-deposits characteristic of Alzheimer disease in retinal scans. Distinctive retinal amyloid deposits are associated with Alzheimer disease, however, their identification requires expert analysis [11]. These two use cases demonstrate the ability of SPECHT to provide adaptive object detection and classification to multi-scale fluorescent microscopy data sets. The kurtosis scaling and belief theory based object identification are not restricted to microscopy use cases, or to the features we use. To the best of our knowledge we are the first to employ belief theory based object identification for WSOD/L, enabling the usage of belief calculus for more general applications, as well as leveraging it to span heterogeneous datasets. By separating the object detection stage from the identification and labelling, we ensure that our approach will detect objects that have no or weak discriminative information for an image level label. This capability is important because it enables a more comprehensive quantitative analysis of images by recording both rare, common, unique, and distinguishing objects in a variety of images. Learning to distinguish common objects in sets of images allows our models to learn to identify those in new data where exactly those objects are altered by disease, dysfunction, or genomic modification. Without decoupling the object detection stage, we would run the risk of overfitting a learned model only on discriminative information in the current set of images. The unbiased identification also enables frequency analysis, where we measure the change in frequency of ‘discriminative’ objects with respect to ‘common’, rather than being bound to counting of discriminitative objects alone.

## Method

In this section, we outline the design of our proposed method. To help the reader unfamiliar with some of the domain specific terms we have 3 glossaries of imaging (S1 Table in S1 Appendix), belief theory (S2 Table in S1 Appendix), and biology (S3 Table in S1 Appendix) related terms to help understanding of the contribution and ensure terms are unambiguously defined.

### Adaptive kurtosis aligned object detection

#### Object detection principle

**Fig 2:**
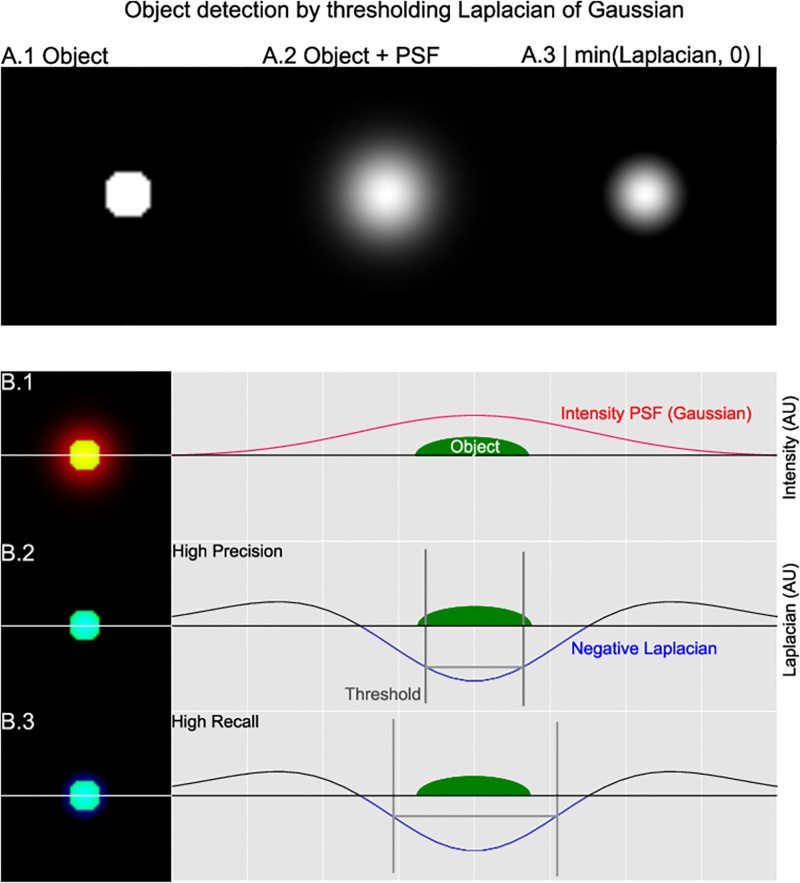
Object detection principle. The negative Laplacian (A.3-V, B.1)) can be leveraged to detect Gaussian 2D observations of 3D fluorescent objects. Thresholding V is a balance between high precision (B.2) and high recall (B.3)

While simple manual thresholding can balance a trade-off between precision and recall, finding the same consistent balance across images, channels, and datasets using manual thresholding requires a per-image threshold and is sensitive to operator variance. The image Laplacian ∇^2^, a measure of the second derivative of the image intensity, can be used to detect edges of objects wherever ∇^2^ changes sign. In 2D microscopy images of 3D fluorescent deposits, we can leverage that connected components of *V* = |min(∇^2^, 0)| (Alg. 1, line 5) coincide with the approximate outline of the objects, since the intensity curve of such observations is bell-shaped (Fig 2) when the fluorescent marker is labelling complex spherical structures with a non-constant height.

Non-specific binding can, given its tendency to self-organise [51] in concentrations of fluorescent label, can have a similar intensity profile. More formally, the domain, use case, and acquisition allow us to state that the intensity profile for a single object can be approximated by a generalized normal distribution with probability density function $\frac{\beta }{2\alpha \Gamma \beta^{-1}}{\text{e}}^{{-\frac{\left|x-\mu \right|}{\alpha }}^{\beta }}$ with scale *α*, location *μ*, the gamma function *Γ*, and 1 ≤ *β* ≤ 3. We apply a 2-stage Gaussian (Alg. 1-line 4) smoothing before and after *V* to ensure pixellation effects are minimized, with *σ* set at or below the precision of the system. This is related to the Laplacian of Gaussian (LoG) approach, underlying ‘blob’ detection in, for example, ‘scale-space’ object detection [52]. However, in the classical computer vision formulation of ‘blob’ detection, the object representation is assumed to have a constant or similar representation, not bell-shaped as is the case in our fluorescent microscopy use cases. The 2^nd^ *σ* is used to smooth rectilinear effects by the Laplacian operator. The first can be omitted when the acquisition microscopy has a specialized deconvolution operator tuned to the imaging point spread function.

#### Self-tuning adaptive object detection

**Fig 3:**
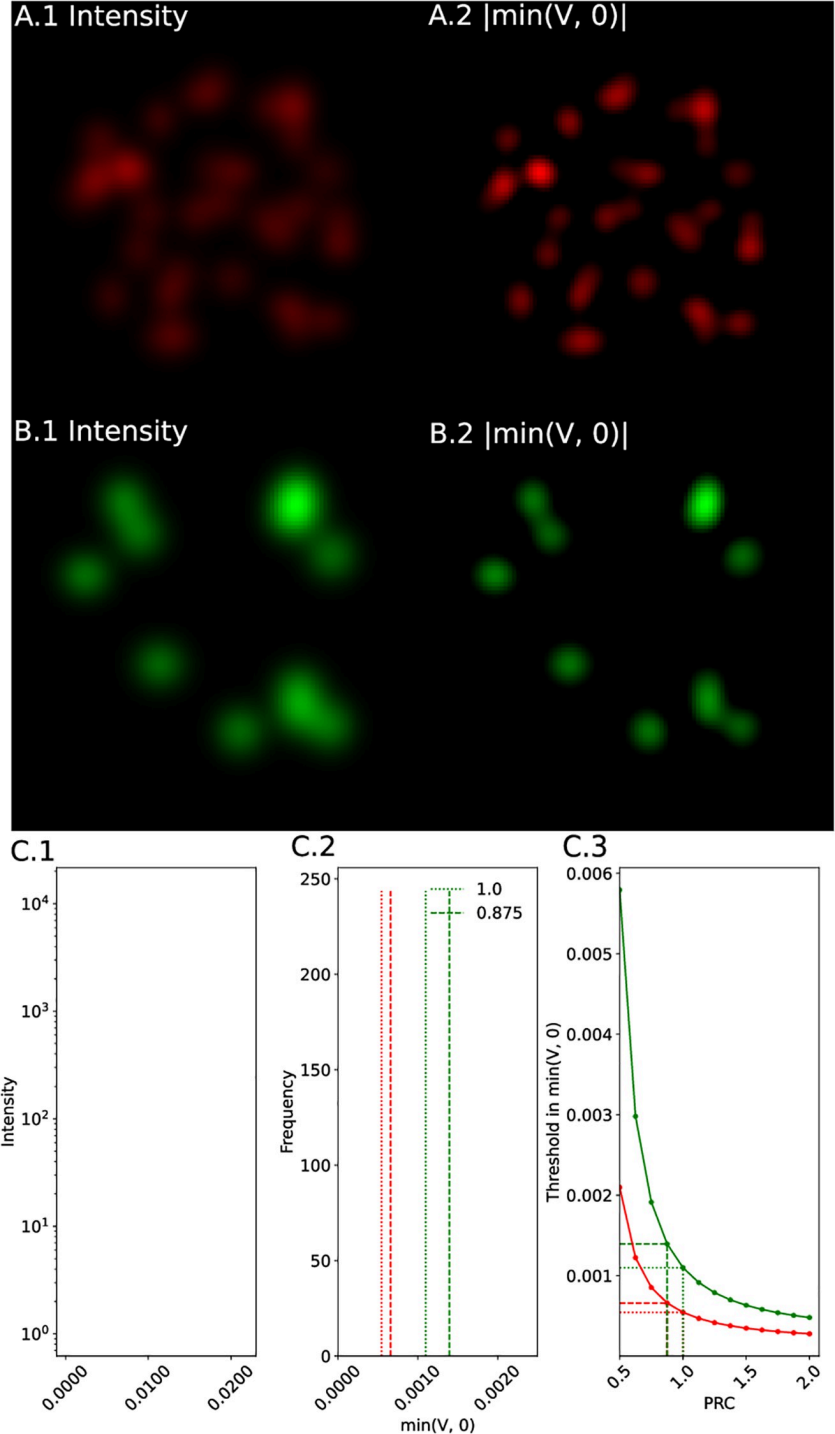
Adaptive thresholding. Kurtosis based thresholding illustrated on two in silico images. A: N = 35, $\sigma =\frac{10+5\times \text{r}and()}{3}$, B: N = 12, $\sigma =\frac{15+5\times \text{r}and()}{1}$, sources randomly placed, with isotropic PSF. A.2 and B.2 show the negative Laplacian, and illustrate how it is less susceptible to intensity differences. C.1: The intensity distribution of both images. C.2: The distribution of the Laplacian of both images. C.3: The automatically derived threshold based on kurtosis can be scaled in favor of precision (PRC < 1) or recall (PRC > 1). The plot shows how kurtosis space thresholding follows the different shapes of the distributions.

Given an object detector that gives a higher response with respect to the location of the object, we need to threshold the response to obtain a binary mask serving as object detection. To unburden the practitioner and improve reproducibility as well as consistency across images and channels, a self-tuning approach is needed. The practitioner can be given the option to express their intent in favoring precision or recall (Fig 3), in terms of object retrieval, and expect to have that intent translated consistently across heterogeneous datasets into corresponding values in the parameter space of the object detection method.

In order to express user intent consistently, we have to find a way to translate parameters across distributions of *V*-space (negative Laplacian). We observe that an image with a few bright objects will have a long-tailed distribution in *V*-space, whereas an image with a high frequency of faint objects will have a short-tailed distribution in *V*-space (Fig 3 red, green, respectively). The kurtosis of a distribution is a scalar value increasing with the ‘tailed-ness’ of the distribution. We illustrate this by means of in silico data (Fig 3), where the threshold in kurtosis space scales with the shape of the two different distributions (Fig 3C.2-green versus red). An increase in frequency of objects in an image will lead to higher V values, and with them a change in the tailed-ness of the distribution. Conversely, a decrease will lead to a shorter tailed distribution, given that most V values are caused by the image background. If we can find a thresholding method that adapts to the tailed-ness of the distribution of V, then we are more likely to obtain consistent across images. We next use these insights to normalize *V* to $Z_{V}=\left|\frac{V_{i}-E\left(V\right)}{\sqrt{\text{Var}(V)}}\right|\;\forall \;V_{i}$ and then obtain an estimate $E_{z}^{\prime }∼E\left[Z\right]$ (the expected z-score, or ‘standard’ score), as a consistent threshold, that can be scaled up or down automatically across images. While we can compute E[Z]=∫zf(z)dz, this entails that we have a probability density function, which in practice involves fitting a parametric function, a process that is non-trivial to do consistently across datasets, and unless corrected, will have a larger error at the tails of the distribution. Due to inaccurate estimation we can end up with estimates that for one image underestimate E[Z], yet for another overestimate. We then have results that vary per image in precision and recall, rather than being consistent in their results. If we aim for a lower bound on E[Z] then we maximize consistency. If a preference for precision over recall is preferred, one can weight the lower bound, while retaining consistency across images. We can derive such a strict lower bound by noting that $\text{kurtosis}\left(V\right)=E\left[Z_{V}^{4}\right]$[53]. By a special case of the Cauchy-Schwartz inequality, we know that

$$\begin{array}{c} \forall x_{i}\in R^{+}\;\;\sum_{i=1}^{n}x_{i}^{2}\leq {\left(\sum_{i=1}^{n}x_{i}\right)}^{2}\leq n*\sum_{i=1}^{n}x_{i}^{2}\;\text{if}\;n<\infty \end{array}$$ (3)

from which it then follows that $\sum_{i=1}^{n}Z_{i}^{4}\leq {\left(\sum_{i=1}^{n}Z_{i}\right)}^{4}$. We can then derive:

$$\begin{array}{c} \sqrt[{4}]{k(X)}\leq E\left(Z\right). \end{array}$$ (4)

We now have a lower bound approximation $E_{z}^{\prime }$ to E(Z) that allows us to express a threshold on the normalized Laplacian that scales with the shape of the distribution of the negative second derivative of the image, producing consistent results across images, channels and datasets. We use the ‘excess’ kurtosis (*k*-3) in our implementation. Moreover, by weighting the kurtosis, we can allow the user to alter the threshold in a distribution-aligned space. We scale the outcome by a floating point parameter ‘precision-recall (PRC)’ to fulfill our aim of an intent-based self-tuning and adaptive method. A value PRC >1 leads to a distribution-aligned object extraction that favours recall, PRC ≤1 favours precision. Fig 3 illustrates the scaling effect on in silico distributions. However, our results illustrate the need for an auto-tuning approach where the object detection method retrieves objects consistent with the end-user intent by aligning the distributions of image differentials.

We note that even though we apply the kurtosis scaling on the negative Laplacian, there are no constraints to extending this approach to multinomial distributions of arbitrary features, as long as those have finite moments. Computing kurtosis is non-parametric, e.g. does not expect a certain family of distribution, hence easily generalizes to new applications.

Finally, we binarize the image where a pixel is 1 if and only if the corresponding negative laplacian exceeds the dynamic threshold. The binarized image is then decomposed by using the connected components algorithm treating the 2D image as a graph. The complete algorithm to detect objects from a heterogeneous set of images is listed in Alg. 1.

**Algorithm 1** Adaptive kurtosis-based self-tuning object detection

1: **Input** Set 2D images **I**, parameter *σ*_1_, *σ*_2_, precision-recall ratio (PRC)

2: **Output** Binary object masks *M*

3: **for** *I*_*j*_ ∈ **I** **do**

4:  $\nabla_{j}^{2}\leftarrow {\text{Gaussian}}_{\sigma_{1}}(\text{Laplacian}\left({\text{Gaussian}}_{\sigma_{2}}\left(I_{j}\right)\right)$

5:  $V_{j}\leftarrow \left|\text{min}\left(\nabla_{j}^{2},0\right)\right|$

6:  $k_{j}\leftarrow E{\left(\frac{V_{j}-\mu_{V_{j}}}{\sigma_{V_{j}}}\right)}^{4}$           ⊳Kurtosis, 4th moment

7:  $z_{j}\leftarrow \sqrt[{4}]{k_{j}}$             ⊳Adaptive consistency across channels

8:  $V_{j}\left[V_{j}\leq \mu_{g}\left(V_{j}\right)*\sigma_{g}{\left(V_{j}\right)}^{\frac{z_{j}}{\text{PRC}}}\right]\leftarrow 0$     ⊳Eq 4

9:  *M*_*j*_ ← connectedcomponents(*V*_*j*_)

10: **end for**

### Probabilistic object labelling using belief functions

The previous section gives us a function D (Eq 1) that decomposes an image *I* with label *L* into objects *o*. Here we aim to find a function S (Eq 2) that quantifies the evidence for the proposition *o* → *L* for each object.

#### Computing support for an image level label using belief theory

We model the problem of finding S for a label L∈L and image *I*:

$$\begin{array}{c} S_{L}:\left(o\to L\right)\mapsto \left(p,q,r\right)\;\;|\left\{o\mapsto L\right\}\subset \Theta ,o\subset I,\;p,q,r\in \left[0,1\right]. \end{array}$$ (5)

The triplet (*p*, *q*, *r*) follows the notation of Dempster [54] where *p* expresses the belief supported by probabilistic evidence that *o* supports the label *L*. *q* is the belief *o* does not support L. *r* is the uncertainty in measuring the respective beliefs. More formally a belief function on a set of propositions Θ is a function Bel: 2^Θ^ ↦ [0, 1] such that Bel(Θ) = 1, Bel(∅) = 0, and $\text{Bel}(\overset{n}{\underset{i=1}{⋃}}A_{i})\geq \sum_{I\subset \{1,..,n\}\wedge I\neq \subset }{\left(-1\right)}^{|N|+1}\text{Bel}\left(A_{i}\right)\;\;\;\forall A_{i}\subset \Theta .$

**Fig 4:**
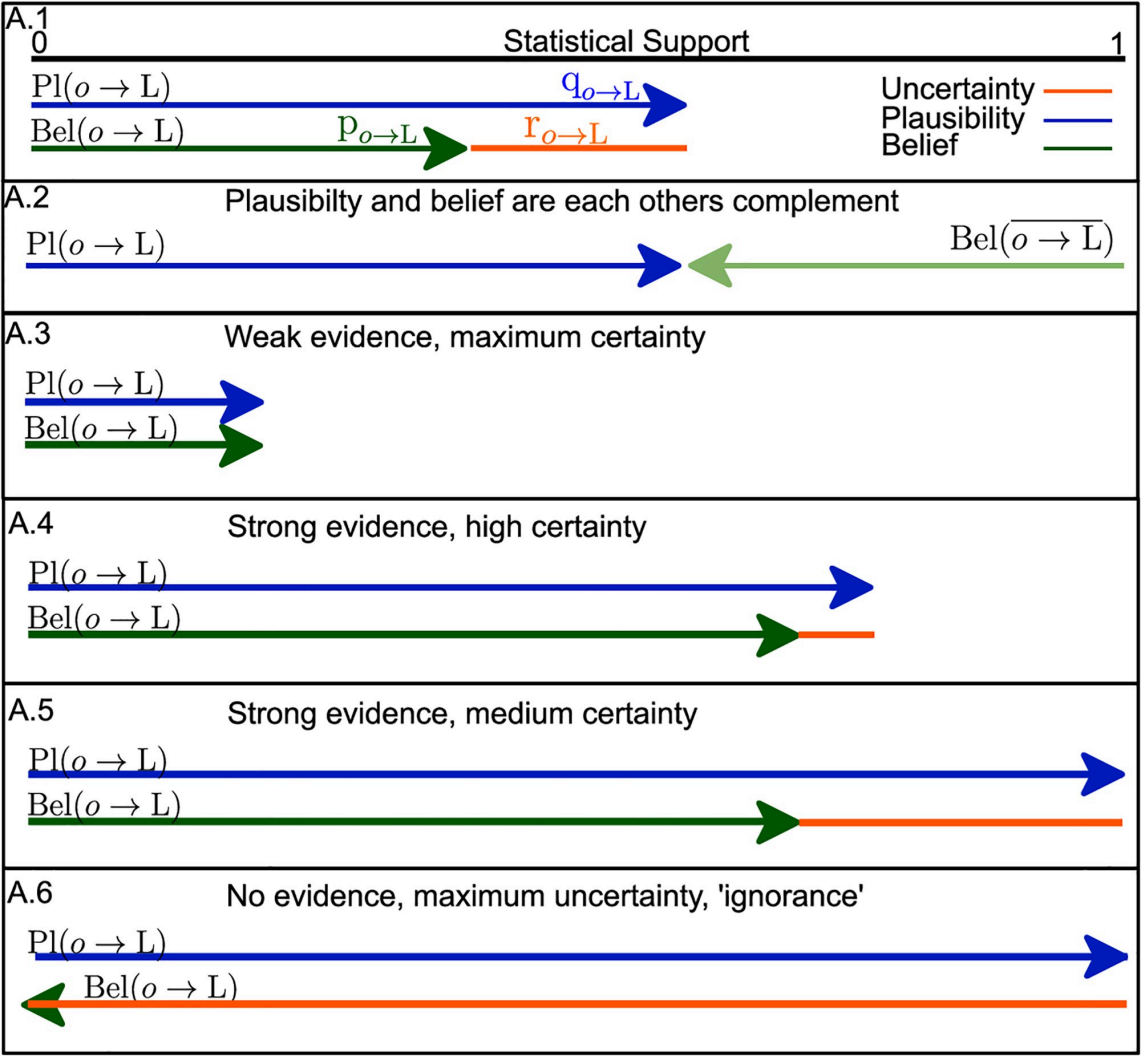
Belief theory. Graphical illustration of the concept of plausibility, belief, and uncertainty in the context of belief theory and as used in the remainder of this manuscript. A.2: Plausibility and belief can be expressed as the complement of their respective support. A trivial, or naive, model has a plausibility of 1, belief 0, and uncertainty 1. A.3–6: Illustrates the flexibility of belief theory based modelling. Weak, but certain evidence (A.3) occurs when belief and plausibility are equal, yet small. Conversely strong evidence can be certain (A.5), but does not need to be (A.4). Finally, absence of quantifiable evidence is mapped to ‘ignorance’, maximal uncertainty, where belief is 0, plausibility1.

Evidence can be encoded by a mass function *m*(*A*)→[0, 1]|*A* ⊂ Θ, where subsets A are referred to as ‘focal elements’, such that $\sum_{A\subset \Theta }m\left(A\right)=1$. Probability functions and probabilities in Bayesian inference are a special case of belief functions where all focal elements are singletons. Unlike probability functions, for general belief functions $\text{Bel}\left(\bar{A}\right)\neq 1-\text{Bel}\left(A\right)$. The ‘plausibility’ function is defined as $\text{Pl}\left(A\right)=1-\text{Bel}\left(\bar{A}\right)$, and Pl(*A*)≥Bel(*A*)∀*A* ⊂ Θ. In the (*p*, *q*, *r*) notation, we have that $p=\text{Bel}\left(A\right),q=\text{Bel}\left(\bar{A}\right),r=\text{Pl}\left(A\right)-\text{Bel}\left(A\right)$. The reader can find a graphical illustration in Fig 4. For a more in-depth review of belief theory, we refer the interested reader to Yager et al. [55].

#### Encoding evidence

Given a set of images **J** with label *L*_*J*_, and a set of images **I**, we want to identify objects in the images and assign to each object *o* a tuple (*p*, *q*, *r*) expressing the belief, plausibility and uncertainty of the proposition *o* → *L*_*J*_ for objects in images in **I**. We illustrate in the results section that our method can be applied to distinguish objects from a nested hierarchy *L*_*J*_ ⊂ *L*_*B*_ ⊂ *L*_*A*_. In Alg. 2, we illustrate the steps we undertake to arrive at a belief based labelling of objects in images. The sets of images **J** and **I** can originate from different channels. The adaptive object detection stage ensures consistent results regardless of channel. After object detection (Alg. 1), we compute a feature descriptor for each object; in our experiments: intensity (sum), area (pixel count) and Laplacian (*V*, sum), a simple, low-dimensional, with non-independent features. We next compute the statistical distance of any object *o* to the distribution of objects in images **J** in feature space using the Mahalanobis distance (Alg. 2-line 11) which accounts for co-dependent dimensions. The Mahalanobis distance range ([0, ∞)) is not interpretable as a mass function.

#### Inferring plausibility

We want to be able to quantify both relative support and support for an individual label. Since the Mahalanobis distance has a range [0, ∞), we normalize the statistical distance (Alg. 2, line-13) so we can leverage Cantelli’s theorem [56]

$$\begin{array}{c} \text{Pr}\left[Z_{i}\geq z\right]\leq \frac{1}{1+z^{2}} \end{array}$$ (6)

to assign a theoretical upper limit to the probability that the object in question supports a label, which then becomes the plausibility *q*_*j*_ = Pl(*o* → *L*_*J*_)≥Bel(*o* → *L*_*J*_). From belief theory [54], we know that $\text{Bel}\left(\bar{A}\right)=1-\text{Pl}\left(A\right)$. For *o* ⊂ I we can formulate $p_{i}=\text{Bel}\left(\bar{o\to L_{J}}\right)=1-q_{j}$. When we swap **I** and **J**, we can obtain *q*_*i*_ and *p*_*j*_, giving us *r*_*i*_ = *q*_*i*_−*p*_*i*_ and *r*_*j*_ = *q*_*j*_−*p*_*j*_. Fig 8C illustrates the application of belief theory based labelling on object detection and the interplay between belief and plausibility (Fig 4A). The resulting support function has no limiting specific priors or assumptions, is continuous, has a theoretical upper bound, and requires no supervised training data. When we are interested in relative support, comparing support for *L*_1_ versus *L*_2_, the statistical distance can be sufficient without normalization. However, normalization allows us to compute plausibility and support for individual labels. Fig 4A1–4A6 provides a graphical illustration of the flexibility of the belief theory framework, and can help the reader understand the definitions of ‘uncertainty’, strong versus weak ‘evidence’, and ‘ignorance’ or absence of information.

**Algorithm 2** Probabilistic labelling algorithm

1: **Input** Images **J** with label *L*_*J*_, Images **I**

2: **Output** Pl_*I*_, plausibility labelled objects for **I**

3: *M*_*I*_ ← objectdetect(**I**, *σ*_1_, *σ*_2_, *PRC*)     ⊳Alg. 1

4: *M*_*J*_ ← objectdetect(**J**, *σ*_1_, *σ*_2_, *PRC*)     ⊳Adapts to channel

5: *F*_*J*_ ← {features(*o*_*j*__*i*_) |*o*_*j*__*i*_ ∈ *M*_*J*_[*j*], *j* ∈ [1, |**J**|]}

6: $\mu_{J}\leftarrow E\left(F_{J}\right),\;\Sigma_{J}\leftarrow \text{Cov}\left(F_{J}\right)$

7: *D* ← [ ]

8: **for** *I*_*j*_ ∈ **I** **do**

9:  **for** *o*_*k*_ ∈ *M*_*i*_[*j*] **do**

10:   *x*_*k*_ ← feature*s*(*o*_*k*_)

11:   $D_{j}\left[k\right]\leftarrow \sqrt{{\left(\vec{x_{k}}-\vec{\mu_{J}}\right)}^{T}\Sigma_{J}^{-1}\left(\vec{x_{k}}-\vec{\mu_{J}}\right)}$     ⊳Mahalanobis

12:  **end for**

13:  $Z_{j}\leftarrow \frac{D_{j}-E}{(}D_{j})\sqrt{\text{Var}(D_{j})}$     ⊳Z-normalization

14:  **for** *o*_*k*_ ∈ *M*_*i*_[*j*] **do**

15:   $\text{Pl}\left[o_{k}\to L_{J}\right]\leftarrow \frac{1}{1+Z_{j}{\left[k\right]}^{2}}$     ⊳Eq 6

16:  **end for**

17: **end for**

Next we apply our method to 2 use cases. First, we show how to apply our method on a hierarchical problem formulation where we differentiate between 3 nested labels {*o* ∈ *L*_*CAV*1*KO*_}⊂{*o* ∈ *L*_*PC*3_}⊂{*o* ∈ *L*_*PC*3−*CAVIN*1_} where a subset label is more specific as illustrated in Fig 8B). We validate our results with independent biological ground truth and previous art. We offer a parameter sensitivity study to quantify robustness. Second, we illustrate how to extend our method across heterogeneous small datasets and compute a joint belief function while quantifying the conflict between the composite belief functions. While belief theory based combination has been used for histopathology classification [57], our usage for individual object detection in microscopy is to the best of our knowledge novel.

#### Using belief calculus to express joint models spanning heterogeneous data

Especially in the case of human tissue data of patients, data is sparse and usually acquired by different institutions, with operators, acquisition, and protocols varying. Using a single sparse dataset degrades statistical power. Here we show how a practitioner can leverage belief calculus to compute an interpretable joint model over such datasets. We use Dempster’s combination rule [58]:

$$\begin{array}{c} m\left(A\right)=\frac{\sum m_{1}\left(B\right)m_{2}\left(C\right)|B\cap C=A}{\sum m_{1}\left(B\right)m_{2}\left(C\right)|B\cap C\neq \subset },\;|A\subset \Theta . \end{array}$$ (7)

to define a joint belief function that combines the evidence from both sources to support a proposition A (*o* → *L*), while allowing the expression of the disagreement. Dempster’s rule uses probability mass functions, which we can obtain from our belief functions by observing that our propositions (*o* → *L*) are singleton focal elements, therefore in our case Bel(A) = m(A), with $\text{m}\left(A\right)=\sum_{B\subseteq A}{\left(-1\right)}^{\left|A\backslash B\right|}\text{Bel}\left(B\right)$. We enumerate in Table 1 the intermediate results needed to compute the joint mass function for our use case. Let for a proposition *A* = (*o* → *L*) the probability mass $m_{H_{1}}\left(A\right)=t$ and $m_{H_{2}}\left(A\right)=s$ respectively. The table is indexed by subsets of all propositions (Θ) on which the belief functions are defined. An entry in the table on row B, column C represents $m_{H_{1}}\left(B\cap C\right)*m_{H_{2}}\left(B\cap C\right)$. The joint mass function *m*_*H*^′^_(*A*) is then given by:

$$\begin{array}{c} m_{H^{\prime }}\left(A\right)=\frac{ts}{1-((1-s)t+(1-t)s))}. \end{array}$$ (8)

Combining sources of evidence should be accompanied by a quantification of their disagreement or conflict to allow a practitioner transparency in the construction and usage of the joint model. The weight (W) of conflict of the joint mass function, an expression of the disagreement between the two models, is given by the logarithm of the normalisation term W = −log(1−((1−*s*)*t* + (1−*t*)*s*))). Combination is not meaningful when both sources are in complete contradiction, that is (*t*, *s*) = (0, 1)∨(1, 0). In such cases, W is infinite, allowing the practitioner a sanity check. However, probability values of exactly 0 or 1 are extremely unlikely in practice. The formulation of a closed form expression for the joint model, allows us to span or ‘fuse’ models across heterogeneous data. Using Dempster’s combination rule to combine models has been shown to be a robust method to combine multiple heterogeneous object detector models on natural images (‘Dynamic Belief Fusion’) [59], where it outperformed both Bayesian fusion and weighted sum approaches. In addition, the application was detection of discrete classes of objects in a supervised setting, e.g. detecting a ‘chair’ in a natural image. More importantly, the computation and reporting of conflict was not leveraged, as is the application across heterogeneous datasets. These are important distinctions with respect to applications in microscopy, where object types are fuzzy or continuous, and heterogeneous data adds further complexity to the fusing of models, as well as necessitating the reporting of conflict to the end user.

**Table 1:** Dempster combination enabling the expression of a joint model. *A*, *B*, *C* ⊂ Θ.

*B*∩*C* → [0, 1]	$m_{H_{1}}$ (*A*) = *t*	$m_{H_{1}}\left(\bar{A}\right)$ = 1−*t*
$m_{H_{2}}$ (*A*) = *s*	*A* → *ts*	∅ → *s*(1−*t*)
$m_{H_{2}}\left(\bar{A}\right)$ = 1−*s*	∅ → (1−*s*)*t*	$\bar{A}\to \left(1-s\right)\left(1-t\right)$

In the following section, we will apply our method to two distinct use cases to illustrate more advanced usage, in addition to validating the method.

## Results

Next, we evaluate SPECHT on in silico, and real world data. The full description of the real world datasets used in this section is listed in the S1 Appendix (Sec. S5 Text in S1 Appendix). Each subsection has a detailed breakdown of dataset structure, as this differs per use case. The use cases share that each is composed of 2D image / label pairs, where each image is a 2D observation of 3D fluorescent labelling.

### Evaluation of object detection on in silico data

#### Consistency across datasets

**Fig 5:**
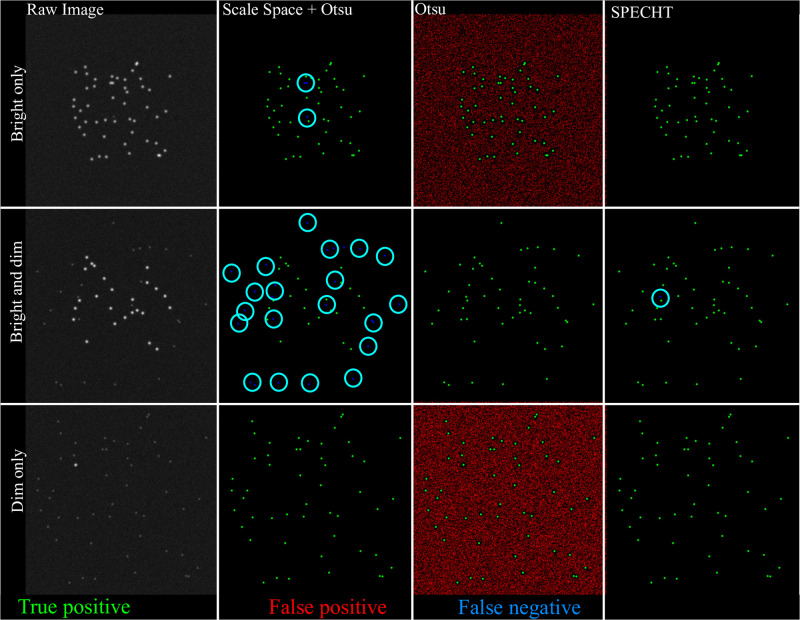
Consistency compared to existing methods. We simulate 3 markedly different in silico scenarios where light sources are either dominated by bright, dim, or are a mixture of both. Note that SPECHT is not always optimal, but does produce consistent results across these variable conditions. Green marks true positives, the location of the actual objects. Red marks false positives, predicted objects that do not exist. Blue circles denote false negatives, objects that should be detected, but are not.

In order for the belief labelling stage to function with minimal bias, it is critical that the object detection stage performs consistent, and predictable across datasets. It is indeed possible to design or train a method to perform optimal on a single chosen dataset, but compromise performance to an unknown extent on future datasets. A major source of variance across datasets, in fluorescence microscopy, is the distribution in size and brightness of labelled objects. Variance of fluorescence is not only a common obstacle across datasets, but also appears in multiple channel analysis, given that two fluorescently marked targets are rarely exhibiting the same distribution, even in the same cell. We need this consistency, given that we have high variance both across cells and channels, in our real world datasets. We simulate images of 512 × 512 pixels, with a Gaussian and Poisson noise model [60] of respectively *σ* and λ set to 0.062 (in 8-bit grayscale). In each image, k bright and j dim objects are randomly placed, where k ∈[1, 25, 50], and j ∈[50, 25, 1]. Bright objects are modelled with a Gaussian PSF (*σ* = 3), whereas dim objects have *σ* = 6 and with their intensity reduced by a factor of 4. For our specific use case, where no ground truth is available and little domain knowledge can be exploited, we compare against 2 tried and true approaches: automatic scale space detection [52, 61], and Otsu thresholding [62]. More advanced object detection methods have become available for fluorescence microscopy [63, 64], but these invariably require parameters related to the objects of interest, e.g. scale range. In our use case we do not have that information, and estimating it would risk propagating size-based biased information to the belief based labelling. The scale space algorithm is pre-configured with the range of *σ*’s of objects to detect, which in our real data is not available. In addition, the output of the scale space detection is filtered by an Otsu-filtering stage to remove false positives. SPECHT’s PRC is set to 2, with *σ* set to the PSF *σ*. Fig 5 illustrates the results. Objects are considered correctly reconstructed when the detected object overlaps with the ground truth (green). False positives are marked in red, false negatives in blue. Under these varying conditions, SPECHT is not always optimal (middle row, Otsu), but is very consistent in object retrieval. In contrast, the two reference methods can be optimal for a given distribution, but vary markedly. If domain specific information is available, or ground truth data, more targeted object detection stages can be used in place of our adaptive method. However, reliance on a consistent, adaptive object detection stage provides the capability to obtain similar results on future unseen datasets, without having to worry about potential parameter sensitive bias.

#### Robustness to noise

**Fig 6:**
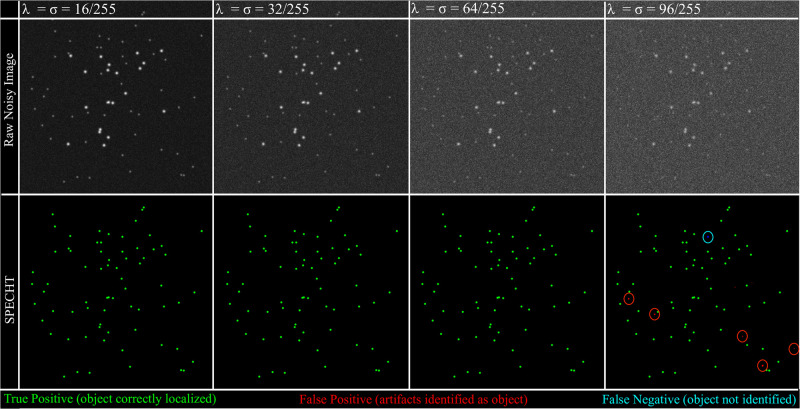
Sensitivity to noise. SNR decreases rapidly as the parameters of both noise sources (Gaussian, Poisson) are increased, yet SPECHT’s recovery of faint objects remains stable under moderate noise conditions. At severe noise levels, as is expected, artifacts appear, while sources with intensity lower than background intensity can no longer be recovered. Green marks true positives, the location of the actual objects. Red marks false positives, predicted objects that do not exist. Blue circles denote false negatives, objects that should be detected, but are not.

Noise from different sources in unavoidable in fluorescence microscopy data. While the Laplacian operator is sensitive to noise, the classical sequence of Gaussian-Laplacian-Gaussian mitigates noise amplification by smoothing. However, at severe noise levels the smoothing step itself can introduce artifacts, that then lead to false positives or skew the Laplacian operator output. By pruning SPECHT’s output with a local maxima heuristic, we can mitigate introduction of false positives. Recovery of signal that is below background noise is infeasible. To measure the effectiveness of our algorithm under increasingly noisy conditions, we simulated a mixture of bright and dim light sources (Gaussian PSF), then added both Gaussian and Poisson noise. In Fig 6 we see that at severe levels of noise (*σ* = λ = 96/255, or 0.37 in 8 bit grayscale images), SPECHT starts to introduce false negatives and omit faint light sources. However, note that even at intermediate values (*σ* = λ = 64/255), recovery of faint objects is not compromised. Robust object detection is highly relevant to our application, given that fluorescence labelling can vary across targets, channels, and datasets. In ideal settings, one would preprocess data with specifically developed denoising algorithms, but it is nonetheless important to measure SPECHT’s sensitivity to low signal-to-noise ratios (SNR).

#### Robustness and consistency in real STED microscopy images

**Fig 7:**
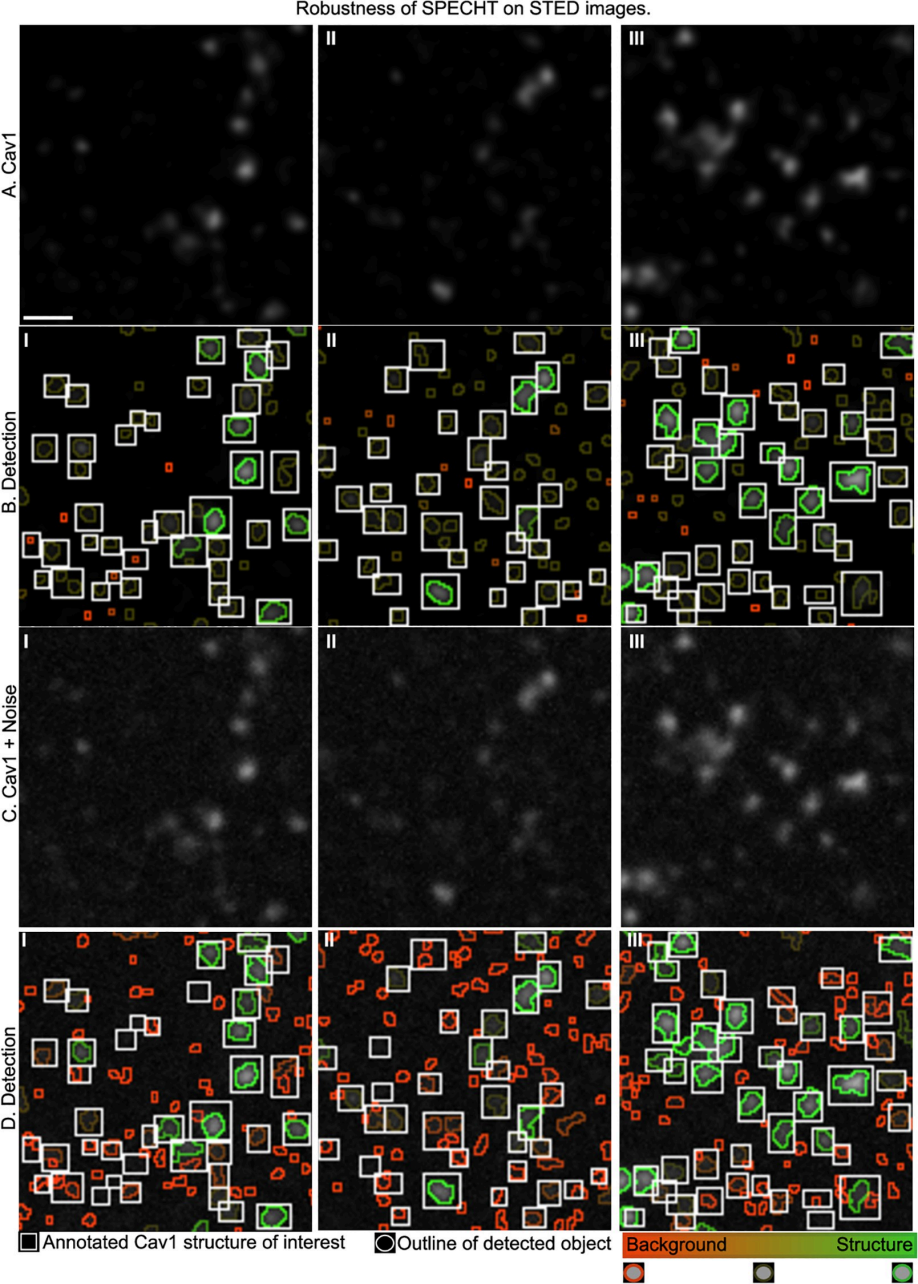
Robustness on partially annotated STED images. We illustrate how the belief stage of SPECHT complements the object detection stage on real world STED microscopy images (A.I-III), subsequently degraded with severe noise (C). We first run SPECHT in high recall mode on selected ROIs of a PC3-CAVIN1 cell, where an expert makes partial annotation (B-white box) of structures of interest. The belief stage then uses a single CAV1 KO cell to learn which identified objects are non-specific labelling (red), versus actual objects of interest (green), on a continuous scale. Next, we repeat the experiment but severely degrade the image with added Poisson and Gaussian noise. The reduced signal to noise ratio induces more artifacts, but the belief stage identifies these as non-specific labelling with high plausibility. Scale bar (A.I) = 120nm

Recent work [65] showed that real world variation in the acquisition of fluorescence microsopy can mislead analysis into concluding that changes at the subcellular level are being recorded, instead of acquisition induced changes. To test SPECHT’s robustness on real STED images to this kind of variation, we had one expert (TW) annotate 3 ROIs of a PC3-CAVIN1 image, drawing bounding boxes on CAV1 structures of interest. Annotation was done blinded to object detection results by SPECHT. We run both the detection and identification stage of SPECHT. The object detection stage is run in high recall mode (PRC 4.25), to recover any potential object. Next, we use the identification stage to discriminate between objects with a signature typical of background and actual CAV1 structures. The results are visualized in Fig 7A and 7B. Detected objects are visualized by their outline, colored by a belief label that scales from background (red) to structure (green). Here, background refers to cytosolic labelling [38]. To further test the robustness, we only use 1 PC3-CAVIN1 and 1 CAV1 KO image to construct the model, compared to the 3×30 images in our other experiments. Annotation is done by bounding box using ImageJ [66]. We then apply noise (Gaussian and Poisson) to the same images, reusing the annotations. In other words, while SPECHT will see only noisy images, the expert is saved the degradation. We repeat detection and identification, with results visualized in Fig 7C and 7D. Note how the addition of noise clearly introduces artifacts when noise exceeds the intensity of faint objects. The identification stage is equally capable of picking up on this change and labelling these new false positives as background. The severity of the noise also induces some of the medium size faint objects to change label towards background, as their intensity no longer is distinguishable from objects typical in CAV1 KO cells. Note that each object is scored on a belief scale, given that we are capturing objects whose class labels are not discrete, rather, are expected to fall on a spectrum of continuous construction and deconstruction dynamics from Caveolae to scaffolds and vice versa with intermediate stages present. SPECHT’s intended deployment is to quantify such dynamics, and thus a discrete classification would induce unacceptable loss of information. The unknown ground truth, the large number of objects (in the order of 50,000 per image), and the inter-annotator variability limits the utility of user annotation. Nevertheless, manual annotation remains valuable baseline to compare against.

### Capturing the gradual construction of complex protein structures in STED

**Fig 8:**
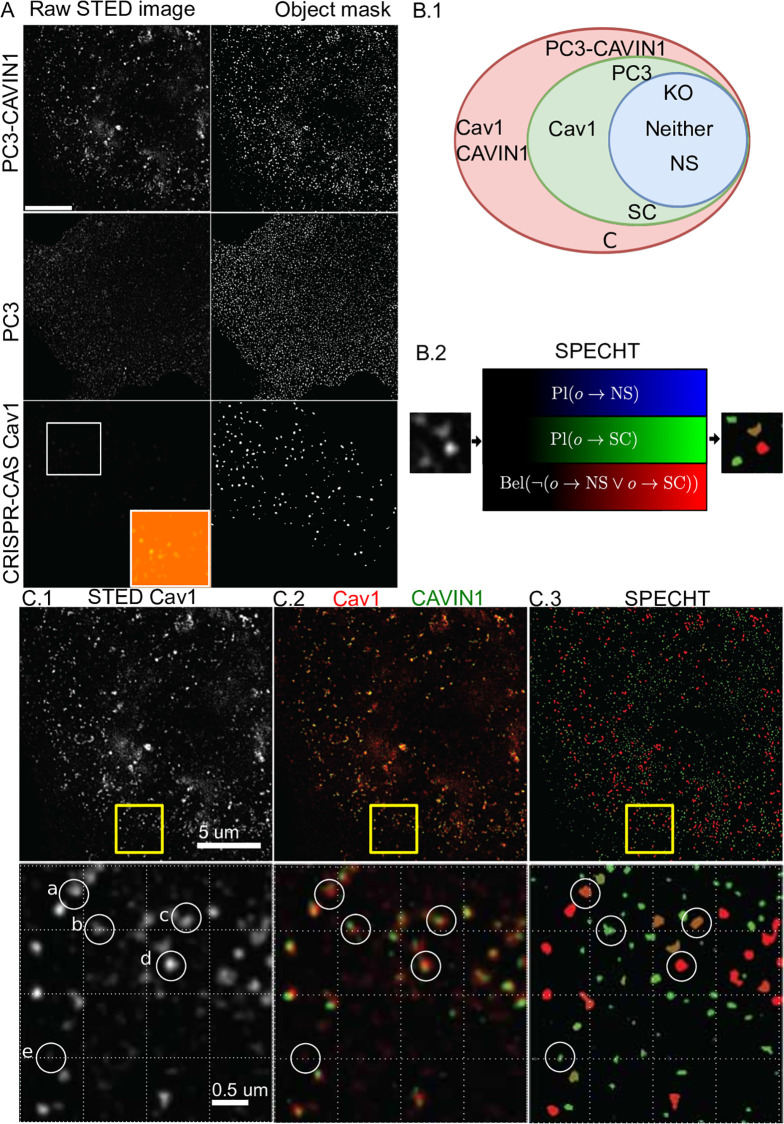
Visualization of results on CAV1 datasets. A: Object detection results on the 3 cell lines with a markedly different intensity profile. B.1: A Venn diagram illustrating how we differentiate between different genotypes. B.2 SPECHT labelling function assigns each object 3 values representing the belief that the object is evidence for either of the 3 object types. C: Illustration of the results on a PC3-CAVIN1 cell. C.3-a, c, d are identified as caveolae with high likelihood, C.3-b as scaffold, C.3-e as background.

CAV1-labelled fluorescent deposits are identified in STED microscopy images and assigned a belief label describing where the identified concentration is on the spectrum between non-specific background labelling (BG, Fig 8C), scaffolds (SC), or caveolae (C). BG deposits are fluorescent markers not associated with their biological target CAV1 molecules. BG can be considered background signal, but is differing from signal perturbing noise. BG fluorescent marker can have remarkable self-organising properties similar to free floating proteins [51]. Identifying BG allows us to exclude it from our biological targets. We study 3 cell lines: CAV1 CRISPR/Cas KO MDA-MB-231 cells with genetically disabled expression of CAV1, PC3 with genetically disabled expression of CAVIN1, and PC3-CAVIN1 with CAVIN1 and CAV1 enabled [67]. In CAV1 KO we can only observe BG, in PC3 only SC and BG, in PC3-CAVIN1 the SC, BG and C are present (Fig 8B). Our label space L is then {BG, SC, C}, with subsets PC3={BG, C} and PC3-CAVIN1 = PC3∪{C}.

#### Experimental procedure

We detect fluorescent deposits (Alg. 1) in CAV1 KO and PC3 cell images and apply the belief function labelling (Alg. 2) to obtain *q*_*x*_ = Pl(*o* → *L*_*x*_) and $p_{\bar{x}}=\text{Bel}\left(\bar{o\to L_{x}}\right)=1-q_{x}$, where *x* is BG, PC3 respectively. Next, we process superresolution images of fluorescence labelled CAV1 deposits in PC3-CAVIN1 (shorthand P3) cells. PC3 cells contain both BG and SC objects, or more formally *q*_PC3_ = *q*_SC_ + *q*_BG_, therefore *q*_SC_ = max(*q*_PC3_−*q*_BG_, 0). The max formulation ensures the correct assignment of 0 plausibility, when outlier objects have values *q*_*PC*3_ < *q*_*BG*_. The subtraction of plausibility functions represents the elimination of the maximum support of a subset (BG) from a superset (PC3) to correctly bracket the maximum support of the subset SC = PC3\BG.

We know that objects unique to PC3-CAVIN1 cells are (formations of) caveolae (C), therefore $p_{\text{C}}=\text{Bel}\left(o_{\text{P3}}\to L_{C}\right)=\text{Bel}\left(\bar{o_{\text{P3}}\to L_{BG}}\right)\wedge \text{Bel}\left(\bar{o_{\text{P3}}\to L_{\text{SC}}}\right)=p_{\bar{\text{BG}}}*p_{\bar{\text{SC}}}$. We visualise the results for a single PC3-CAVIN1 cell in Fig 8C where blue, green, and red gradients correspond with *q*_BG_, *q*_SC_ and *p*_P3_, respectively. From visual inspection, we see correlation of colocalized CAVIN1 with objects labelled with a high *p*_P3_ value, as expected (Fig 8C.3.a, d).. More interestingly, we can now identify objects that are transitioning between SC and C (Fig 8C.3.c). To confirm this, we next perform extensive validation.

#### Validation

**Fig 9:**
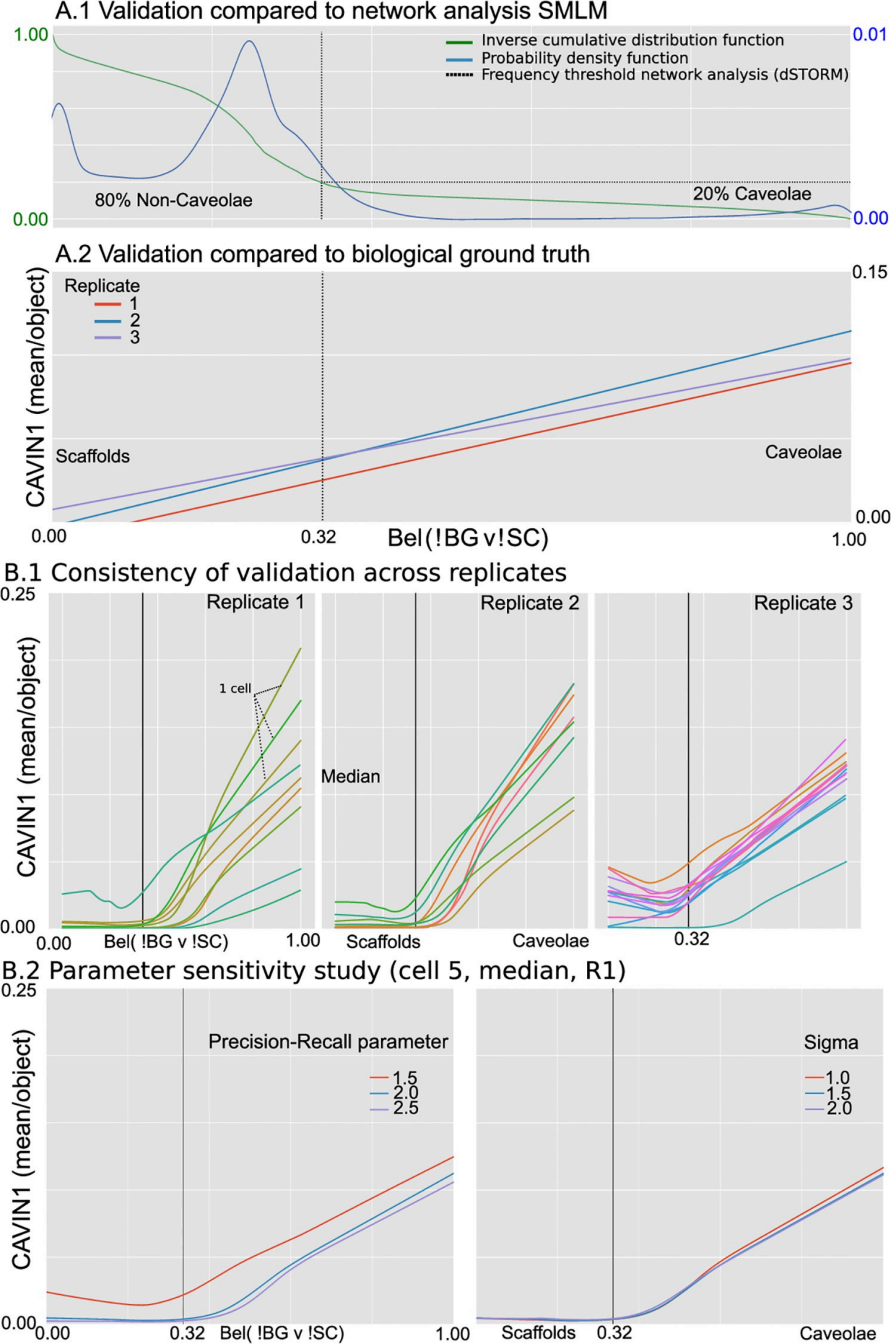
Results on CAV1 dataset. A: Validation with respect to previous art (A.1) and biological ground truth (A.2). A: The distribution of SPECHT’s label (A.1, x-axis: P[object] = caveolae) shows a distinct long left tail, containing 20% of the data. The frequency division matches previous art in dSTORM analysis. Caveolae only form in the presence of CAVIN1, therefore the probability of an object being Caveolae should correlate with the colocalization of CAVIN1 (A.2), which is what we observe. B.1: The detection threshold (∼0.35, A.1–2) matches the sudden rise in colocalization when we use a LOWESS regression, rather than a linear regression, and results are consistent across 3 replications (30 cells total, each line represents a single cell). B.2: Varying hyperparameters does not alter the consistency of the result with respect to biological ground truth (colocalization CAVIN1). The dotted line corresponds to the frequency (20%) of Caveolae detected in dSTORM using network analysis.

Given that there is no object-level ground truth available, a direct evaluation is impossible, nor is there to the best of our knowledge a method that discriminates between caveolae and scaffolds in 2D STED. Therefore, the only feasible validation is using previous work on caveolae detection in dSTORM, and colocalization of CAVIN1, essential for formation of caveolae, two independent sources of information, not leveraged during the design of the method. First, we know from previous art that the frequency of caveolae in the PC3-CAVIN1 cell line has been reported at ∼20% [5], when compared to other CAV1 structures. SPECHT computes a belief (*p*_C_) for each detected object that it likely is a caveolae. In Fig 9A, we show the cumulative distribution function (cdf) of that belief function. We observe a trimodal distribution, as expected for each of the 3 labels (C, SC, BG). The label distribution shows a long left tail, corresponding with 20% of the data, demarcated at the sudden rise of the cdf (*p*_C_ ∼0.32), matching a transition into the 2nd mode of the trimodal distribution. In other words, if we threshold the belief label at 0.32, the point where the belief function splits into major and minor part, we find the exact same frequency of caveolae-like objects as previously reported. Second, we know that caveolae can only form in the presence of CAVIN1. Therefore, we expect to see an increasing correlation of CAVIN1-CAV1 colocalization as *p*_*P*3_ increases. We compute CAVIN1 colocalization *P* by measuring the mean CAVIN1 colocalization intensity for each CAV1 object. The regression computes a linear model between *p*_P3_ and *P* for all objects, for all cells, per replicate (Fig 9B, replicate is a repeat experiment to ensure consistency). CAVIN1 colocalization increases markedly when *p*_P3_ increases. In Fig 9B a LOWESS-regression [68] is computed to discover a more nuanced behavior in the correlation with CAVIN1 association. All cells show a consistent pattern across replicates. More importantly, the belief value where the colocalization of CAVIN1 suddenly increases, matches the threshold found when comparing to previous work (0.32), confirming the belief function is consistent with biological ground truth and prior work. The SPECHT color legend is overlaid for ease of interpretation. In conclusion, SPECHT’s label indicating an object is likely to be caveolae is consistent with the expected frequency of caveolae in PC3-CAVIN1, and the colocalization of CAVIN1.

#### Parameter sensitivity study

Our method has 2 parameters: the Gaussian *σ* (std, Alg. 1) used in the smoothing and the precision-recall balance. Sigma should be at or below system precision to avoid creating artificially joined objects. For the CAV1 dataset, we omit the first Gaussian filter (*σ*_1_, Alg. 1), the sigma reported here is *σ*_2_. In superresolution microscopy, a deconvolution operation tuned to the acquisition specific point spread function is more accurate in restoring the signal. PRC is set at the user’s discretion; it is nonetheless important to document what its exact impact on the result can be. In Fig 9B.2 we compute the results for replicate 1, Cell 5, the median of the trend (Fig 9B1.1). A lower PRC (1.5) results in fewer, brighter objects dominating the selection. Fewer spots similar to non-specific CAV1 binding will be included, explaining the upward shift of the curve while retaining the trend. When PRC is high (2.5) the inverse process occurs with BG spots driving the mean CAVIN1 association lower. A larger sigma (2 ↔ 1) can lead to low intensity borders being included into the mask of an object. When those pixels are outside the actual caveolae structure the expected CAVIN1 association is not that of caveolae but of background, reducing the mean CAVIN1 association, resulting in lowered correlation. We conclude that our parameter space does not invalidate our results with the two independent sources of information. Our method is therefore capable of extracting and identifying CAV1 structures in STED superresolution microscopy.

### Identifying retinal amyloid-*β* deposits associated with Alzheimer disease

We illustrate how we can extend our method for measuring A*β* across three heterogeneous sparse datasets of fluorescence confocal microscopy images of retinal cross-sections after A*β*-specific immunohistochemistry, acquired using two different microscopes, each operated by a different researcher. Rather than counting objects in the image, we use the belief function to identify which fluorescent marker deposits are more likely to be present in an AD image.

#### Applying belief functions to identify AD across heterogeneous data

We collected the following sets of images and labels:

- ${\text{I}}_{H_{1}}$, *L*_*H*_: retinal tissue from healthy donors, microscope 1, n = 2
- ${\text{I}}_{H_{2}}$, *L*_*H*_: retinal tissue from healthy donors, microscope 2, n = 3
- ${\text{I}}_{D_{1}}$, *L*_*AD*_: retinal tissue from AD-confirmed donors, microscope 1, n = 3.

**Fig 10:**
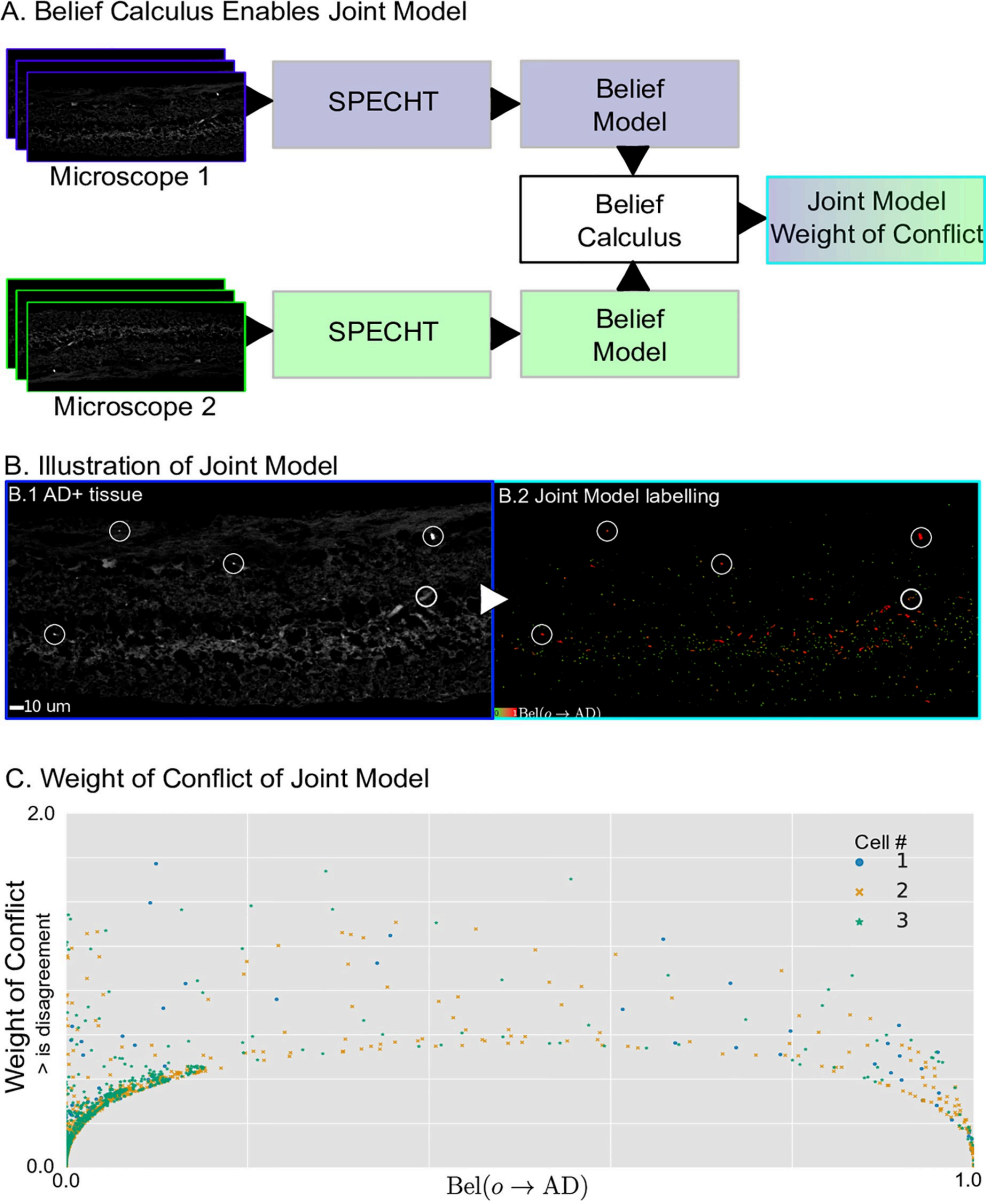
Results on Alzheimer data. A: Belief calculus enables the combination of models learned data originating from different microscopes. B: We visualize how the joint model operates on a single image of retinal tissue stained for amyloid-*β*, sourced from an AD+ positive patient. Object marked in red express a high belief in being AD+ specific. C: We offer the end user a per-object expression of the conflict between the 2 models that create the joint model. An increased weight of conflict (Y-axis) indicates the models disagree on the labelling for a specific object. We illustrate the visualization here for 3 AD+ images. Observe that for objects where both models are uncertain (∼.5) their minimal conflict is higher than it is for objects that have a higher support for being either AD+ specific or healthy.

We show an example AD+ image in Fig 10B.1. We identify fluorescent objects in all healthy images using Alg. 1 and obtain $q_{x_{L_{H}}}=\text{Pl}\left(o\to L_{H}\right)$ where x indicates which set of healthy images is used (1,2). Next, for each object detected in each AD image, we obtain as before $p_{\bar{x_{L_{H}}}}=1-q_{x_{L_{H}}}=p_{x_{L_{\text{AD}}}}$.

In Fig 10 we illustrate the idea and visual results as well as the quantification of conflict that is offered to the end user. The individual belief functions are consistent in their results with respect to each other and the visually easily observable AB-deposits. The joint belief function combines both models to offer a weighted combination of the evidence provided by each model. In Fig 10C we plot the weight of conflict of the joint belief function for all 3 AD images. The weight of conflict is the smallest at both extrema of the joint belief function, indicating that the models from the two different microscopes agree the most for the objects that are strongly believed to originated from a healthy or AD retina by the joint belief function, while there is a greater disagreement for the objects without strong belief. A practitioner can use the weight of conflict for each object-prediction pair to quantify the agreement between multiple sources of evidence along with the output of joint evidence based on the joint belief function.

## Discussion

The motivation for this work was the need for a robust, adaptive, and self-tuning unsupervised probabilistic object detection method applicable to heterogeneous multi-scale superresolution microscopy. While it is feasible to use a larger number of more elaborate features to describe objects, e.g. deep learning, during development we found using simple low-dimensional features and statistical modelling obtained results validated by biological ground truth. We note that our formulation of belief functions makes them separable (*A*∩*B* is a focal element) and consonant (*A* ⊂ *B* or *B* ⊂ *A*) support functions [14]. As a result, our *p* and *q* functions are equivalent to ‘necessity’ and ‘possibility’ functions from possibility theory [69]. We note that the joint model can also be formulated when objects from 2 different models overlap, for example, when we run our method with different *σ* and PRC values to obtain two models, one with high recall, one with high precision. In such a joint model, we now have for each object *o* an inner, smaller object *o*’. One formulation for focal elements then can be: *A* = {(*o* → *L*)∧(*o*^′^ → *L*)}, *B* = {(*o* → *L*)∧¬(*o*^′^ → *L*)}, leading to a more complex formulation for a joint model. A more interesting use case is when the object detection is fuzzy and allows for non-empty intersections. Due to space constraints we discuss the computation of uncertainty in S6 Text in S1 Appendix. The belief theory framework allows us to work with nested or hierarchical labels, as well as leverage the mathematically sound concepts of conflict and joint models. The object detection stage is designed to be robust to long tail distributions and high variations in density.

### Limitations and future work

When the intensity profile of the fluorescence diverges from a generalised normal distribution, our object detection will increasingly fail and split objects into parts; a different detection method is then warranted [70]. In addition, when image quality degrades to low signal to noise values (SNR), the intensity distribution can cause negative adjusted kurtosis values. In this case raising such a negative value to a fractional power is a domain error. A deconvolution or task specific denoising is recommended to recover or improve SNR before analysis, and will typically be part of an image processing pipeline. The Mahalanobis distance can be uninformative in high-dimensional space due to the ‘curse’ of dimensionality, however, this is only the case if the increase in dimensions is due to non-discriminatory features [71]. While the joining of belief functions by Dempster’s rule is not without criticism [72], we note that the preconditions [72] for its use are satisfied in our case with independent evidence sources and exclusive exhaustive hypotheses. In future work, we aim to adopt advances in evidence combination [73] to enable quantification of reliability of individual sources and make the joint model robust against unreliable sources. We are in the process of extending the approach to incorporate temporal information to more accurately capture and reconstruct biological construction dynamics, and identify which temporal dynamics are conditional on weak nested labels. In addition, the exploration of multiple channels, as well as fusing information from multiple domains offers more information that can be exploited not only to increase discovery, but to improve the performance of stages of the method itself. Finally, the usage of the conflict formulation can be explored with human experts in the loop, to explore its usefulness as an interpretable tool. We refer the interested reader to S6-S8 Texts in S1 Appendix for a more in-depth discussion on uncertainty and numerical stability, as well as sensitivity to noise.

## Conclusion

We introduced a novel adaptive self-tuning method for object detection in 2D microscopy images of fluorescent labelled proteins that enables consistent results across channels, and a novel method to assign each object a belief that expresses numerically the evidence encoded. We validated our method on superresolution data of CAV1 deposits, where we showed agreement with related work and biological ground truth. We showed we are able to identify and characterize CAV1-labeled caveolae and scaffolds by STED superresolution microscopy, setting the stage for robust, reproducible temporal live cell analysis where consistency across images and channels is essential for scientific discovery. We applied our method on an Alzheimer pilot study, illustrating the multiscale applicability. We illustrated with a closed form expression the capability to formulate a joint model spanning heterogeneous datasets while recording the conflict of evidence between the separate models as a reliability measure.

## Supporting information

S1 Appendix (PDF)
