# Supplementary material for: SPECHT: Self-tuning Plausibility based object detection Enables quantification of Conflict in Heterogeneous multi-scale microscopy
Source: PLoS One. 2022 Dec 29;17(12):e0276726. doi: 10.1371/journal.pone.0276726 (PMC9799313; doi:10.1371/journal.pone.0276726)
Supplement: S1 Appendix — (PDF) [file pone.0276726.s001.pdf]

S1. Table Imaging terms glossary.

**Term**	**Description**
I	2D fluorescence microscopy image
L	Image level label (e.g. image of patient with Alzheimer positive diagnosis: L = AD+)
$\overline{L}$	The complement of the label L, for example if L=AD+, then $\overline{L}$ expresses that the patient is healthy.
$\mathcal{L}$	The label space, superset of all labels, defined by the domain.
$D(I)$	Function decomposing an image into objects ($o \subset I$) based on their fluorescent marker
o	Detected object, a set of adjacent pixels, in image I ($I = \{\cup o \mid \forall o \subset I\}$)
∇^2	Image Laplacian, measure of the second derivative of image intensity.
V	The absolute value of the negative part of the image Laplacian: V=$\|\min(\nabla^2, 0)\|$
σ	The standard deviation of a normal (Gaussian) distribution, used here as a parameter of Gaussian (smoothing) filter.
Z_V	Normalized negative Laplacian $Z_V = \|\frac{V_i - \mathbb{E}}{(}V)\sqrt{\text{Var}(V)}\| \; \forall \; V_i$
PRC	Precision ReCall, parameter used to scale object detection in our algorithm consistently across channels. A higher value (>1) favors recall, a lower value favors precision
STED	Stimulated Emission Depletion, a superresolution microscopy technique, amenable to live cell imaging.
PSF	Point Spread Function, the measured response or intensity distribution in 2/3D of an imaging system to a light source (point). A common mathematical model, in absence of device specific information, is the Gaussian function.
Deconvolution	In our context, using precise knowledge of the imaging system's PSF in combination with a deconvolution restores (improves) image quality.

S2. Table Statistics and belief theory glossary.

Term	Description
$k(X)$	Kurtosis of the distribution of a random variable X: $k(X) = E[(\frac{X-\mu}{\sigma})^4]$. Kurtosis of a normal distribution is 3, "excess" kurtosis is k-3. k(X) increases as outliers are more extreme and frequent (longer tail of the distribution)
L	Categorical label assigned to an image. A single image can have multiple labels assigned.
$\mathcal{L}$	Set of all subsets of all labels.
$\overline{L}$	Set-complement of the label L, with respect to a label space $\mathcal{L}$. $\overline{L} = \mathcal{L} \backslash L$. E.g. in the AD+ use case, $\overline{AD+}$ = healthy, however for more complex label spaces the complement is not equal to the negation.
$S_L(o)$	Function computing the statistical support, or evidence e the object o has for the image level label L ($o \subset I$)
$Bel(o \rightarrow L)$	The minimum statistical support, or belief, an object o has for the image level label L.
$p_L(o)$	Shorthand for the belief function $Bel(o \rightarrow L)$
$\text{Pl}(o \rightarrow L)$	The maximum statistic support, or plausibility, an object o has for the image level label L
$q_L(o)$	Shorthand for the plausibility function $Pl(o \rightarrow L)$
$r_L(o)$	The uncertainty in evidence based support of object o for label L: $r_L(o) = q_L(o) - p_L(o)$. See Fig. 4 for graphical illustration of the relation of uncertainty to evidence.
Θ	'Frame of discernment' $\Theta = \{o \rightarrow L \mid o \subset \text{I}, L \in \mathcal{L}\}$
Focal element	Subset $f \subset \Theta$ such that f does not contain any smaller subsets of Θ.
cdf/pdf	The cumulative distribution function (cdf) of a random variable X: $F(x) = P(X \leq x)$ and the probability density function (pdf) as the derivative of F(X): $\int_a^b f(x)dx$.
Evidence	Statistical information that can be leveraged to compute statistical support. See Fig. 4 for graphical illustration of strong versus weak evidence, in the context of uncertainty.
Conflict	The disagreement the statistical measures (e.g. belief functions) have on the support that an object o has for an image level label L.
Weight of conflict (W)	A numerical value measuring the disagreement (conflict) between multiple belief functions.
Combination	Given 2 or more belief functions, computing the joint (combined) support as well as conflict that a single object o has for image level label L.
Separable support function	Support function where for all focal elements A, B holds that $A \cap B$ is a focal element.
Consonant support function	Support function where for all focal elements A, B holds that $A \subset B$ or $B \subset A$.

S3. Table Biology glossary.

**Term**	**Description**
CAVIN1	Cavin-1 protein, necessary to form Caveolae.
PC3	Human prostate cancer cell line, here we use the shorthand PC3 to refer to the cells with CAVIN1 expression disabled.
PC3-CAVIN1	PC3 with expression of CAVIN1 enabled.
CAV1	Caveolin-1 protein, main component of Caveolae.
Caveolae (C)	Sub-cellular structures composed of CAV1 protein complexes, ∼100 nm invaginations in the cell membrane.
Oligomer	Molecule consisting of a few identical units, in our contexts proteins, e.g. CAV1 oligomer contains several CAV1 proteins.
Scaffolds (SC)	Non-caveolar scaffolds (SC), including 8S oligomers that can combine to form larger non-caveolar hemispherical scaffolds as well as caveolae.
Non-specific labelling (BG)	Fluorescent marker that is not marking its intended target, contributing to semantic "noise".
KO	'Knockout', a cell line where a particular gene's expression is disabled using the CRISPR/Cas technique.
CAV1 KO MDA-MB-231	MDA-MB-231 is a breast cancer cell line, here with CAV1 expression disabled (KO).
Amyloid-β	A protein whose excess deposits in neuron cells have a complex role in expression of neurodegenerative disease, such as Alzheimer.

S4 Text Code and datasets SPECHT is written as a Julia module leveraging the high level features this scientific programming language offers in combination with high performance. Datasets and code are released under open source license (CC By SA 4.0, Affero GPLv3 respectively) at `https://github.com/bencardoen/SPECHT.jl`.

S5 Text Data acquisition and cultures. PC3, PC3-CAVIN1 and CRISPR/Cas CAV1 KO MDA-MB-231 cells [1-4] were cultured in RPMI-1640 medium (Thermo-Fisher Scientific Inc.) complemented with 10% fetal bovine serum (FBS, Thermo-Fisher Scientific Inc.) and 2 mM L-Glutamine (Thermo-Fisher Scientific Inc.) at 37 Celsius in a 5% CO2/95% air incubator. Cells grown on 1.5H coverslips (Paul Marienfeld) were fixed with 3% paraformaldehyde (PFA), 15 min at room temperature, rinsed with PBS, permeabilized with 0.1% Triton X-100 in PBS plus 0.1 mM Ca2+ and 1 mM Mg2+ (PBS-CM) and blocked with 10% Goat Serum (Thermo Fisher Scientific, Waltham, MA) and 1% bovine serum albumin (Sigma, St. Louis, MO) in PBS-CM. Cells were incubated with the primary antibody (12h, 4 Celsius) and the secondary antibody (1h, room temperature). The primary and secondary antibodies were diluted in SSC (saline sodium citrate) buffer containing 1% BSA, 2% goat serum and 0.05% Triton X-100. Coverslips were mounted with Prolong Gold (Life Technologies, Thermo Fisher Scientific). Cells were washed after antibody incubations using SSC buffer containing 0.05% Triton X-100. Images were acquired with a 100x/1.4 Oil HC PL APO CS2 STED White objective of a Leica TCS SP8 3x STED microscope (Leica, Wetzlar,

Germany) equipped with a white light laser, HyD detectors, time-gated fluorescence detection and Leica Application Suite X (LAS X) software. Acquisition was done at a scan speed of 600 Hz with a line average of 5. Pixel size is 20nm and resolution (precision) is around 70nm for the CAVIN1 channel and 50nm for the CAV1 channel. GFP was excited at 488 nm and depleted at 592 nm. Alexa Fluor 647 was excited at 653nm and depleted at 775 nm. Huygens Professional software (Scientific Volume Imaging, Hilversum, NL) was used to deconvolve STED images, chromatic aberration correction was applied on CAVIN1 images using the CAV1 channel as reference channel using the 'correlation full' method. Confocal microscopy images of retinal cross-sections after immunohistochemistry staining for amyloid-β. Tissues were obtained from control eyes from Eye Bank of BC, and AD eyes from donors with post-mortem neuropathological diagnosis of Alzheimer's disease from UBC Department of Neurology. Tissues were processed as paraffin embedded cross-sections (5 um). BA4 primary antibody was used for specific binding for the first 2 amino acids of the AB peptide amino terminus, Cy3 secondary antibody was used to label BA4 in red fluorescence. Samples imaged at 543 nm wavelength using Zeiss LSM 510 at 0.44 um x 0.44 um pixel dimension over 450 um x 450 um area, and Zeiss Axio Imager M2 at 0.454 um x 0.454 um pixel dimension over 624.70 um x 501.22 um area. LSM 510 images were resized to match the pixel dimension of those from Axio Imager M2. Images were manually segmented for the retinal layers, the vitreous and the region posterior to the outer nuclear layer were masked to reduce artefactual signals.

S6 Text Discussion on uncertainty in context of belief theory. In this section, we briefly discuss the computation of 'r' (Eq. 5), the uncertainty in measuring the belief. For an object o, label x we have the plausibility $q_x = \text{Pl}(o \rightarrow L_x)$ and belief $p_x = \text{Bel}(o \rightarrow L_x) = 1 - q_{\overline{x}}$. Uncertainty, in this context, is defined as $r_q = q_x - p_x$ Intuitively this makes sense, one can interpret belief as the measurable support, whereas plausibility is the maximum potential support. When we divide the label space $\mathcal{L}$ into 'supports x' versus 'does not support x', we have that $\mathcal{L}$ is composed of two focal elements, x and $\overline{x}$. N ote that while here x is a single label, $\overline{x}$ is not. Examples where $|x| == |\overline{x}|$ are when x is 'healthy' versus 'disease'. Given q_x, and our division of label space (and frame of discernment) into x and $\overline{x}$ we can compute $p_{\overline{x}}$. Finally, we compute $r_x = q_x - p_x$, and $r_{\overline{x}} = q_{\overline{x}} - p_{\overline{x}}$. Our contribution gives us a way to compute both q_x and $q_{\overline{x}}$, therefore we can derive the belief functions, and with them the uncertainty, in essence a 'top-down' computation of belief functions. It is helpful to reflect what this 'uncertainty' actually means for the practitioners. Let us explore the most uncertain scenario, $q_x = 1$, and $p_x = 0$, with $r_x = 1$. Uncertainty increases with the inability of the belief function to obtain evidence ($p_x \rightarrow 0$). An inability to find evidence for a negative ($p_{\overline{x}} \rightarrow 0$), leads to $q_x \rightarrow 1$ and uncertainty increases. Let us now consider the converse, a scenario where uncertainty is minimal. Then it must be that $p_x = q_x$, and $p_{\overline{x}} = q_{\overline{x}}$. This can occur when the information measured is never neutral, the features always support or negate a label, but never both, and our capability to measure those features is perfect. In theory it is possible to obtain such a scenario, by increasing the dimensions of the feature space to infinity. However, one must ensure that the added dimensions (features) are maximizing information (support), otherwise we invoke the 'curse' of dimensionality [5]. A final analogy that can help is that of the balance between precision and recall. Inability to discern (believe in) the true support for a label would lead to low precision, and thus a belief tending to zero for label x. Low recall, conversely, is a belief function unable to discern support for a true negation of the label ($\overline{x}$). Uncertainty informs on both ($r_x, r_{\overline{x}}$, but in our setting is able to infer this without the need for annotation. If this is surprising, consider that quite often objects in an image support both the label x and its negation. When we label an image as 'healthy',

versus ‘diseased’, we can be sure that both labels are supported by a confounded label of ‘tissue’, and perhaps the label of ‘background’ or non-tissue acquisition. The capture of the support for ‘healthy’ will include statistical support that is shared with ‘disease’, because not all tissue is affected equally, and before tissue is diagnosed as ‘diseased’ it has undergone a transition towards ‘diseased’. What we intuitively mean by ‘healthy’, is ‘healthy’ and never seen in ‘diseased’ and vice versa. In practice, this is not expressed in the labels, nor do we tend to encode this in the statistical learning methods employed. In these conditions, uncertainty can help to quantify exactly what we can capture. Due to space limitations and given that it is challenging to validate uncertainty, we have omitted empirical results on computing uncertainty on our datasets as they would be illustrative, rather than quantitative support for our method.

Algorithm 3 False positive filtering heuristic for use in severely degraded SNR images. This is an optional post-processing step using the output of Algorithm 1

Input Raw image I, object mask M, σ)
Output Binary object mask OM
$I_g \leftarrow \text{Gaussian}_\sigma(I)$
$Q1, Q3 \leftarrow \text{quantile}(I_g, [0.25, 0.75])$
$TH \leftarrow Q3 + 1.5(Q3 - Q1)$
$M_I \leftarrow \text{localmaxima}(I_g)$
$OM \leftarrow \text{copy}(M)$
$C \leftarrow \text{connectedcomponents}(M)$
for $M_i \in M_I$ **do**
    $x, y \leftarrow \text{index}(M_i)$
    if $M_i > TH$ **then**
        if $M[x, y] > 0$ **then**
            $c_j \leftarrow C[x, y]$ ▷ Component id
            $OM[C == c_j] \leftarrow 1$
        end if
    end if
end for

S7 Deploying SPECHT in heavily degraded SNR conditions While the kurtosis scaling already guards the object detection stage against perturbations in the presence of noise, in certain acquisition conditions a SNR nearing 1 cannot be avoided due to, for example, non-specific labelling filling the cell, or capturing labelling that is sensitive to the health of its target. In such conditions the error margin of object detection methods diverges quickly leading to large numbers of false positives or false negatives. Achieving consistency under these conditions is non-trivial. Here we document a heuristic that can be used to deploy SPECHT in such conditions. We note that recovering missed objects is not feasible, so the first stage is ensuring maximal recall, by setting the PRC parameter to e.g > 4. In order to then screen out false positives, we can make use of the observation that labelled object in fluorescence microscopy will likely contain a local intensity maxima. In very low SNR conditions we note that the intensity distribution will be dominated, by definition, by the noise values. We can then use these two observations to define a heuristic that detects false positives. We first compute all local maxima on the raw image, optionally preprocessed with a small smoothing factor to reduce an excessive number of local maxima. Given that noise now forms the mass of the intensity distribution, by elimination the local maxima of signal will be found in the outliers of the distribution. We can detect such outliers by computing the interquantile range and setting an intensity threshold

$TH \leftarrow Q1 + 1.5 \times IQR$. We then verify that each detected object contains a local intensity maxima $> TH$. Objects that do not, are removed. As SNR tends to 0 all object detection will fail, but we show that with this heuristic we can use SPECHT on a wider range of SNR values. In addition, consistency across channels is still ensured because the IQR based threshold will scale with SNR. The entire procedure is listed in Algorithm 3.

S8 Text Numerical stability. The closed form expression for combination rule in the Alzheimer use case (Eq. 8) is sensitive to loss of precision due to catastrophic cancellation (loss of significance) when t, s are close to 0, we therefore correct t and s values to max(x, ϵ) with ϵ the machine epsilon. Computing the 4th root of the kurtosis can be numerically unstable. However, we note that the alternative, for example, the geometric mean of V, more appropriate compared to the arithmetic mean given that V is a ratio, has similar issues in that it uses similar operations.

References

1. Khater IM, Meng F, Wong TH, Nabi IR, Hamarneh G. Super resolution network analysis defines the molecular architecture of Caveolae and Caveolin-1 scaffolds. Scientific Reports. 2018;8(1):1–15.
2. Wong TH, Khater IM, Joshi B, Shahsavari M, Hamarneh G, Nabi IR. Single molecule network analysis identifies structural changes to caveolae and scaffolds due to mutation of the caveolin-1 scaffolding domain. Scientific reports. 2021;11(1):1–14.
3. Meng F, Joshi B, Nabi IR. Galectin-3 overrides PTRF/Cavin-1 reduction of PC3 prostate cancer cell migration. PloS one. 2015;10(5):e0126056.
4. Hill MM, Bastiani M, Luetterforst R, Kirkham M, Kirkham A, Nixon SJ, et al. PTRF-Cavin, a conserved cytoplasmic protein required for caveola formation and function. Cell. 2008;132(1):113–124.
5. Zimek A, Schubert E, Kriegel HP. A survey on unsupervised outlier detection in high-dimensional numerical data. Statistical Analysis and Data Mining: The ASA Data Science Journal. 2012;5(5):363–387.
